# Predictive accuracy of genetic variants for eye color in a Kazakh population using the IrisPlex system

**DOI:** 10.1186/s13104-024-06856-y

**Published:** 2024-07-05

**Authors:** Alizhan Bukayev, Igor Gorin, Baglan Aidarov, Akynkali Darmenov, Elena Balanovska, Maxat Zhabagin

**Affiliations:** 1 National Center for Biotechnology, Astana, 010000 Kazakhstan; 2 Research Centre for Medical Genetics, Moscow, 115522 Russia; 3 Karaganda Academy of the Ministry of Internal Affairs of the Republic of Kazakhstan named after Barimbek Beisenov, Karaganda, 100000 Kazakhstan

**Keywords:** Forensic genetics, IrisPlex System, SNP Genotyping, Microarray, Eye color, Kazakh population

## Abstract

**Objective:**

This study assesses the accuracy of the IrisPlex system, a genetic eye color prediction tool for forensic analysis, in the Kazakh population. The study compares previously published genotypes of 515 Kazakh individuals from varied geographical and ethnohistorical contexts with phenotypic data on their eye color, introduced for the first time in this research.

**Results:**

The IrisPlex panel’s effectiveness in predicting eye color in the Kazakh population was validated. It exhibited slightly lower accuracy than in Western European populations but was higher than in Siberian populations. The sensitivity was notably high for brown-eyed individuals (0.99), but further research is needed for blue and intermediate eye colors. This study establishes IrisPlex as a useful predictive tool in the Kazakh population and provides a basis for future investigations into the genetic basis of phenotypic variations in this diverse population.

**Supplementary Information:**

The online version contains supplementary material available at 10.1186/s13104-024-06856-y.

## Introduction

DNA fingerprinting has been widely adopted in the field of forensic science [1]. Over the course of nearly four decades, it has consistently proven its effectiveness in determining an unidentified individual by comparing the DNA profiles of crime scene evidence and potential suspects. Nevertheless, this approach is constrained by the absence of comparative DNA material of the suspect that forensic scientists may not always possess. The issue at hand can now be addressed due to the rapid advancement of forensic DNA phenotyping (FDP) technology. This technology enables the prediction of various physical characteristics, such as age, gender, and other traits, solely based on DNA analysis, even when the individual is unidentified [2]. The accuracy of DNA prediction is currently being investigated in relation to various external characteristics of individuals, including eye color, hair color, skin color, eyebrow shape, presence of freckles, hair structure, male pattern baldness, and height. These characteristics are being actively studied and incorporated into forensic practice [3].

The phenotypic characteristic of eye color is highly notable, as it encompasses a wide range of spectral variations, including darker hues such as brown, lighter shades like blue, as well as mixed or intermediate tones such as hazel, gray, green, and yellow. The observed diversity can be attributed to the genetic regulation of melanogenesis, which is characterized by variations in melanosomes composition and the level of melanin within the iris epithelium [4]. The primary factors influencing eye color are the OCA2 and HERC2 genes, as indicated by previous research [5–7]. The OCA2 gene exhibits polymorphism that is responsible for a significant portion, approximately 74%, of the observed variation in eye color. This genetic variation also includes the presence of the highly abnormal oculocutaneous albinism variant [8]. The transcriptional activity of the OCA2 gene is modulated by the HERC2 gene, which is situated in close proximity [9]. Furthermore, the formation of eye color involves the participation of additional genes, including ASIP, IRF4, MC1R, SLC24A4, SLC24A5, SLC45A2, TYR, and TYRP1 [10]. Several Genome-Wide Association Studies (GWAS) have been conducted to identify the primary polymorphisms associated with the development of eye color phenotypes [11–17]. The IrisPlex system for predicting eye color included the following significant polymorphisms: HERC2 - rs12913832, OCA2 - rs1800407, LOC105370627 - rs12896399, SLC45A2 - rs16891982, TYR - rs139330, and IRF4 - rs12203592 [18]. Various methods have been employed for the prediction of pigmentation traits, including Bayesian logic, classification trees, and alternative sets of SNP variants [19–21]. Nevertheless, the IrisPlex model that combines multiple factors is widely favored in academic circles owing to its user-friendly interface and exceptional predictive accuracy. The accuracy of predictions, exceeding 90%, has been evaluated in diverse populations across various regions worldwide [22–26]. Nevertheless, it should be noted that certain Asian populations exhibited different outcomes [27, 28], possibly due to the fact that the IrisPlex system was initially designed and tested on European populations.

The objective of this study is to evaluate the accuracy of the IrisPlex system for predicting eye color in the Kazakh population. The Kazakhs are one of the Asian populations, controlling vast swaths of land from the Altai to the Caspian Sea. The society of the Kazakh population was organized based on a hierarchical patrilineal system of genealogical lineages, clans, and tribes. These, in turn, formed three socio-territorial groups known as “zhuz”: the Senior zhuz primarily occupied Southern and South-Eastern Kazakhstan, the Middle zhuz resided in Eastern, Northern, and Central Kazakhstan, and the Junior zhuz traditionally lived in Western Kazakhstan. We aim to determine how well this system predicts eye color in the Kazakh population and how it compares to predictions in other populations.

## Methods

The selection of 515 study participants was based on population biobanking criteria [29]. Every participant in the study provided their informed consent by signing a consent form, completing a questionnaire (Additional file 1: Questionnaire), and contributing venous blood samples. The sample under study encompassed five distinct regions of Kazakhstan, namely the north (*N* = 68), south (*N* = 198), center (*N* = 69), west (*N* = 58), and east (*N* = 62). The sample under study exhibits a gender and age composition consisting of 162 female participants and 353 male participants, with mean ages of 22 and 21 years, respectively. The faces of the participants were captured in portrait mode using a Nikon D5100 18–55 mm lens, ensuring consistent lighting conditions and maintaining the same distance. Eye color was divided into three categories, namely blue, intermediate (green/hazel), and brown, by three trained independent investigators. This classification was in accordance with the recommendations provided by earlier studies [18, 28].

The extraction of DNA from venous blood was performed using the Wizard (R) Genomic DNA Purification Kit (Promega, USA) following the manufacturer’s recommended methodology. The genotyping of markers linked to eye color was conducted utilizing a Phenotype Expert kit provided by the DNA Research Center, LLC, located in Russia. Steps in the experiment included multiplex polymerase chain reaction (PCR), hybridization of the amplified PCR result onto a biochip, and then genotype determination. The comprehensive elucidation of the Phenotype Expert kit and technique has been previously furnished [30]. The provided kit comprises a collection of 60 genetic markers that have been identified as being associated with various phenotypic traits such as eye color, hair color, skin color, ABO blood group, sex determination, and core Y-chromosome haplogroups specifically among the Kazakh community. The data note [31] contains the published raw genotyping data for the 60 genetic markers in the 515 Kazakhs. The data accessed on the National Center for Biotechnology Information Reference Assembly dbSNP repository (Build 157 Release) under:

[https://www.ncbi.nlm.nih.gov/SNP/snp_viewTable.cgi?handle=LHG].

The statistical toolset in GenAlEx6 [32] was utilized to perform calculations for allele frequencies, observed heterozygosity (Ho), expected heterozygosity (He), and tests for conformity with Hardy-Weinberg equilibrium (HW) and linkage disequilibrium (LD). The Spearman correlation coefficient and the hypothesis of independence of attributes were assessed using Pearson’s chi-squared test with the assistance of XLSTAT software (https://www.xlstat.com/en/). Eye color predictions were produced in three distinct groups (blue, intermediate, and brown) using the online tool provided by the Department of Genetic Identification at Erasmus MC (https://hirisplex.erasmusmc.nl). The determination of the probability of predicting eye color is conducted through the utilization of a multinomial logistic regression (MLR) model, and a 0.7 probability threshold was employed as recommended by Walsh et al. [33]. The evaluation of the predictive outcomes achieved using the IrisPlex system was conducted in a manner consistent with our prior research [28], employing the subsequent algorithmic quality parameters:

- Precision refers to the ratio of true positive values to the total number of samples assigned by the classifier to a specific class.
- Recall, on the other hand, represents the ratio of true positive values to the total number of samples within that class.
- Accuracy denotes the proportion of data for which the classifier correctly defined the class.
- F₁-measure is a metric that calculates the harmonic mean between precision and recall of the classifier.
- Additionally, AUC (area under curve) indicators are used to evaluate ROC curves. These curves depict the relationship between the proportion of true positive results relative to the total number of samples and the proportion of false positive values relative to the total number of samples, while varying the threshold of the decision rule.

## Results

The results of the genotype distribution of the 6 SNPs (rs12913832, rs1800407, rs12896399, rs16891982, rs1393350, rs12203592) in 515 individuals from the Kazakh population are presented in Additional file 2: Table S1. The frequencies of the alleles and the actual and expected heterozygosity indices are shown in Table 1. Two markers, rs16891982 and rs12896399, exhibited high levels of the heterozygosity index: 0.410 and 0.367, respectively. A marker, rs12203592, exhibited a value of Ho = 0.037, indicating a state that is in close proximity to the monomorphic state. A departure from the state of Hardy-Weinberg equilibrium (*p* > 0.05) was observed in relation to a specific genetic marker (rs12896399). Nevertheless, by implementing the Bonferroni adjustment (*p* > 0.008), the aforementioned deviation is mitigated.

**Table 1:** Allele frequencies, heterozygosity and hardy–Weinberg evaluation of the IrisPlex System in the Kazakh Population City (*N* = 515)

Gene	SNP	Allele Frequencies						HWE
		A	C	G	T	Ho	He	*p*-Value
HERC2	rs12913832	0.840	-	0.160	-	0.270	0.269	0.944
OCA2	rs1800407	-	0.954	-	0.046	0.087	0.087	0.942
LOC105370627	rs12896399	-	-	0.717	0.283	0.367	0.405	**0.032**
SLC45A2	rs16891982	-	0.716	0.284	-	0.410	0.407	0.884
TYR	rs1393350	0.090	-	0.910	-	0.177	0.164	0.086
IRF4	rs12203592	-	0.982	-	0.018	0.037	0.036	0.670

* SNPs out of Hardy–Weinberg expectations (*p* > 0.05);

The iris color of the eyes of 515 individuals in the Kazakh population is documented in Additional file 3: Table S2 and summarized in Table 2, categorized as blue, intermediate, and brown. Within the examined sample, it was observed that the prevailing hue was brown, accounting for 86.21% of the occurrences. The proportion of those classified as intermediate and blue accounts for 12.82% and 0.97%, respectively. In a previous study conducted in rural Kazakhstan, a sample size of 60 individuals was examined to determine the frequency of eye color. The results indicated that the incidence of blue eyes was 3.33%, intermediate eye color was 11.65%, and brown eyes were observed in 85% of the participants [34, 35]. Based on the IrisPlex system’s forecast, the sample being examined revealed a prevalence of 98.25% for brown eye color, while the intermediate color category could not be ascertained. Additionally, the likelihood of blue eye color was estimated to be 1.75%. The results of Spearman’s correlation coefficient (*r* = 0.256, *p* < 0.01) and Pearson’s chi-squared test (χ2 = 69.3, *p* < 0.01) suggest a significant relationship between the actual color variables and the IrisPlex system prediction.

**Table 2:** Frequencies of pairwise distribution of iris color prediction in the IrisPlex system and actual data

IrisPlex	Actual data (iris color)			
	blue	intermediate	brown	total
**blue**	2	6	1	**9**
	(0.39%)	(1.17%)	(0.19%)	**(1.75%)**
**intermediate**	0	0	0	**0**
	(0.00%)	(0.00%)	(0.00%)	(0.00%)
**brown**	3	60	443	**506**
	(0.58%)	(11.65%)	(86.02%)	**(98.25%)**
**total**	**5**	**66**	**444**	**515**
	(0.97%)	(12.82%)	(86.21%)	(100.00)%

Table 3 displays the performance characteristics pertaining to the prediction of iris color in the Kazakh population, utilizing the IrisPlex technology. The AUC (area under curve ROC) prediction accuracy scores were 0.88 for the blue color category, 0.77 for the brown color category, and 0.75 for the intermediate color category. The AUC values of blue and brown eye color in this study are comparatively lower when compared to the inhabitants of Western Europe (0.96 for blue and 0.96 for brown) [36]. Nevertheless, when considering Siberia, the AUC score for blue eye color is 0.57, while the AUC score for brown eye color is 0.56. These numbers are similar to those observed in the Kazakh population, which are equivalent to the values found in the European portion of Russia, where the AUC score for blue eye color is 0.89 and the AUC score for brown eye color is 0.86 [28]. A recall rating of 0.99 is assigned to brown eye color, indicating a high level of sensitivity. The recall score of blue eye color is 0.40, whereas intermediate eye color is not observed. The specificity of intermediate (1.00) and blue (0.99) eye hues is considerably high. However, the presence of false positives for brown eyes significantly reduces this value to 0.11. The F1 parameter’s harmonic mean, which combines precision and recall, demonstrates strong performance in predicting brown eye color. There is a need to do further research in order to identify other genetic markers that can enhance the accuracy of predicting pigmentation phenotypes based on genotype, particularly for individuals with blue and intermediate eye colors.

**Table 3:** Characteristics of performance indicators of the HIrisPlex model for genetic phenotyping of the Kazakh population

Phenotype	AUC	Precision	Recall	F1	Specificity
Brown	0.77	0.87	0.99	0.93	0.11
Intermediate	0.75	0.00	0.00	0.00	1.00
Blue	0.88	0.22	0.40	0.29	0.99

Table 4 displays the AUC (area under the curve ROC) values for the prediction accuracy of several groups of Kazakhs, categorized into three distinct categories. The initial category involves the division of the sample into two distinct groups. The term “4/4 Kazakhs” refers to individuals whose all four grandparents (both grandfathers and both grandmothers) are of Kazakh descent. On the other hand, the term “admixture” is used to describe individuals who have at least one ancestor from a different ethnic group. Within the second category, the sub-ethnic differentiations of Kazakhs are delineated as “4/4 Kazakhs,” which include three zhuzes, socio-geographical regions that have evolved over time. The third category pertains to the five distinct geographical divisions within the territory of Kazakhstan, namely the group referred to as “4/4 Kazakhs”.

**Table 4:** Prediction accuracy of AUC for different groups of the Kazakh population

Category	Group	Sample size	AUC		
			Brown	Intermediate	Blue
**Population**	4/4 Kazakh	465	0.75	0.72	0.87
	Admixture	50	0.77	0.73	1.00
**Zhuz**	Senior	95	0.75	0.70	0.98
	Middle	248	0.74	0.71	0.98
	Junior	110	0.76	0.76	0.54
**Region**	North	68	0.75	0.75	*ND*
	South	198	0.78	0.76	0.77
	Center	69	0.79	0.75	1.00
	West	68	0.71	0.70	*ND*
	East	62	0.67	0.62	0.93

ND - not determined

Upon examining the initial category, it becomes evident that the “Admixture” group’s prediction has greater accuracy across all variations in eye color. The senior zhuz exhibits the most reliable prediction indications within the second group, whereas Central Kazakhstan demonstrates the most accurate prediction indicators within the third category. The group labeled “admixture” with blue color and the region of Central Kazakhstan with blue color exhibited the greatest values of prediction accuracy, with an AUC of 1.00 each. The confirmation of the forecast regarding the blue color was limited to these specific groups. Nevertheless, it is important to consider that the presence of the blue color is infrequently observed in these samples, which can significantly impact the outcome. The junior zhuz with the blue color exhibits the lowest value, as indicated by an AUC of 0.54. The hypothesis regarding the presence of a blue tint lacks empirical validation when considering actual photographic evidence. The accuracy of predictions in northern and western Kazakhstan cannot be determined using the blue hue as a basis, as this color has not been observed in these specific places. The AUC exhibits variation among different groups for the brown color category, ranging from 0.67 to 0.79. Similarly, for the intermediate color category, the AUC ranges from 0.62 to 0.76. The comparison of average AUC values for color shades between the second (zhuz) and third (geography) categories indicates that there are no significant differences observed for brown (0.75 compared to 0.74) and intermediate color (0.72 versus 0.72). However, a notable difference is observed for the color blue, with values of 0.83 versus 0.90. It is advisable that forthcoming research endeavors allocate greater attention to conducting a comprehensive examination of the geographical aspects pertaining to the blue color.

## Discussion

This study introduces new findings on the effectiveness of the IrisPlex system in predicting iris color based on genetic markers, focusing on the diverse ethnohistorical and geographical divisions of the Kazakh population in Kazakhstan. The examination of the association between genotype and eye pigmentation phenotypes has substantiated the efficacy of employing the IrisPlex panel in the Kazakh population. The level of accuracy was found to be lower in comparison to groups of Western Europeans, yet higher when compared to Siberia. Brown eye color is characterized by notably high sensitivity values, specifically a value of 0.99. Previous population genetic studies [37, 38] clearly demonstrate that the metapopulations of Europe, Asia, and Siberia are distinct in their genetic background. They also exhibit contrasting prediction accuracies for phenotypes [39], with significantly reduced accuracy for Siberia [28]. It can be hypothesized that the light eye color, which is occasionally observed among indigenous Siberian populations, is associated with different alleles or genes than those found in Europeans. Recent genome-wide association studies involving nearly 195,000 individuals have identified 50 previously unknown genetic loci for eye color [40]. Therefore, the panel developed based on data from Western Europeans may not accurately predict light eye color in the Kazakh population, considering the origin of several tribes within the Kazakh population from the Altai region, Southern Siberia, and Mongolia. Nevertheless, further comprehensive investigations are necessary to examine blue and intermediate eye colors in greater depth. Special attention should also be given to thresholding, which could improve the classification performance of the IrisPlex model [41]. These studies should involve larger population samples, categorize eye color into more refined gradations, and explore additional markers that can enhance the accuracy of predicting eye color within the population of Central Asia and neighboring regions. It is particularly important to apply digital quantification of human eye color, which reveals greater potential in studying this question [42, 43]. This approach allows for categorization within the brown eye color spectrum [44], which is relevant for Asian populations. However, it is also necessary to increase the number of new genetic markers in the predictive panel. At the same time, it should be noted that the perception of intermediate eye colors varies significantly. For visual inspection, the best results for eye color prediction are shown by a two-category system (blue and brown) rather than three (blue, intermediate, and brown) [45, 46].

## Limitations

The limited size of the study sample (*N* = 515 individuals) drawn from the Kazakh population (16 million) may provide limitations in accurately evaluating the association between the examined SNPs and variations in eye color. It should also be noted that eye color determination was conducted using a classical method, not a digital quantitative method, which imposes limitations on the determination of intermediate eye color.

### Electronic supplementary material

Below is the link to the electronic supplementary material.

**Additional file 1**: Questionnaire

**Additional file 2**: Table S1. The genotypes of 515 individuals from the Kazakh population for the 6 SNPs by IrisPlex System

**Additional file 3**: Table S2. The phenotypes of 515 individuals from the Kazakh population regarding eye color

## Data Availability

The data described in this Data note can be freely and openly accessed on the National Center for Biotechnology Information Reference Assembly dbSNP repository under SNP Submission Batch [https://www.ncbi.nlm.nih.gov/SNP/snp_viewBatch.cgi?sbid=1063555] and Supplementary Materials or from the corresponding author.
