# Supplementary material for: Predictive accuracy of genetic variants for eye color in a Kazakh population using the IrisPlex system
Source: BMC Res Notes. 2024 Jul 5;17:187. doi: 10.1186/s13104-024-06856-y (PMC11227171; doi:10.1186/s13104-024-06856-y)
Supplement: Supplementary file 1 — Additional file 1: Questionnaire [file 13104_2024_6856_MOESM1_ESM.docx]

**PARTICIPANT QUESTIONNAIRE**

| Sex |  |
| --- | --- |
| Date of Birth |  |

| **PLACE OF BIRTH** | **Village - City** | **Area-** | **Region - Country** |
| --- | --- | --- | --- |
| Participant |  |  |  |
| Father |  |  |  |
| Grandfather (from paternal line) |  |  |  |
| Grandmother (from paternal line) |  |  |  |
| Mother |  |  |  |
| Grandfather (from maternal line) |  |  |  |
| Grandmother (from maternal line) |  |  |  |

| **ETHNICITY OF PARTICIPANT** | |
| --- | --- |
| Grandfather (from paternal line) |  |
| Grandmother (from paternal line) |  |
| Grandfather (from maternal line) |  |
| Grandmother (from maternal line) |  |

| **TRIBE AFFILIATION** | |
| --- | --- |
| Tribe |  |
| Clan |  |
| Lineage |  |

| **PATERNAL GENEALOGY** | |
| --- | --- |
| 1 – Father |  |
| 2 – Grandfather |  |
| 3 - Great grandfather |  |
| 4th Great grandfather |  |
| 5th Great grandfather |  |
| 6th Great grandfather |  |
| 7th Great grandfather |  |

|  | ***Tribe*** | ***Clan*** | ***Lineage*** |
| --- | --- | --- | --- |
| Mother |  |  |  |
| Grandmother (from paternal line) |  |  |  |
| Grandmother (from maternal line) |  |  |  |

| **SAMPLE ID** |  |
| --- | --- |

**АНКЕТА УЧАСТНИКА / ЗЕРТТЕУГЕ ҚАТЫСУШЫҒА АРНАЛҒАН САУАЛНАМА**

| Пол / Жынысы |  |
| --- | --- |
| Дата рождения / Туған күні |  |

| **МЕСТО РОЖДЕНИЕ / ТУҒАН ЖЕРІ** | **Село - Город** | **Район** | **Область - Страна** |
| --- | --- | --- | --- |
| Участника / Қатысушының |  |  |  |
| Отца / Әкесінің |  |  |  |
| Атасының / Дедушки |  |  |  |
| Әжесінің / Бабушки |  |  |  |
| Матери / Анасының |  |  |  |
| Нағашы атасының / Дедушки по матери |  |  |  |
| Нағашы әжесінің / Бабушки по матери |  |  |  |

| **ЭТНИЧЕСКАЯ ПРИНАДЛЕЖНОСТЬ УЧАСТНИКА / ҰЛТЫ** | |
| --- | --- |
| Атасы / Дедушка по отцу |  |
| Әжесі / Бабушка по отцу |  |
| Нағашы атасы / Дедушка по матери |  |
| Нағашы әжесі / Бабушка по матери |  |

| **РОДОСЛОВНАЯ / РУЛЫҚ ТЕГІ** | |
| --- | --- |
| Тайпасы (Елі) / Племя |  |
| Руы / Род |  |
| Тармағы / Подрод |  |

| **ГЕНЕАЛОГИЯ ПО ОТЦУ / ЖЕТІ АТА** | |
| --- | --- |
| 1 – Әкесінің аты / Имя отца |  |
| 2 – Атасының аты / Имя дедушки |  |
| 3 - Арғы ата / Прадедушка |  |
| 4 – Баба |  |
| 5 - Тек ата |  |
| 6 - Түп ата |  |
| 7 - Жеті ата |  |

|  | ***Тайпа (Елі)*** | ***Руы*** | ***Тармақ*** |
| --- | --- | --- | --- |
| Мать / Анасы |  |  |  |
| Бабушка (по отцу) / Әжесі |  |  |  |
| Бабушка (по матери) / Нағашы әжесі |  |  |  |

| **НОМЕР ОБРАЗЦА / ҮЛГІНІҢ НОМЕРІ** |  |
| --- | --- |
