# Supplementary material for: Predictive accuracy of genetic variants for eye color in a Kazakh population using the IrisPlex system
Source: BMC Res Notes. 2024 Jul 5;17:187. doi: 10.1186/s13104-024-06856-y (PMC11227171; doi:10.1186/s13104-024-06856-y)
Supplement: Supplementary file 3 — Additional file 3: Table S2. The phenotypes of 515 individuals from the Kazakh population regarding eye color [file 13104_2024_6856_MOESM3_ESM.pdf]

Original Sample ID	Eye Color			
	Blue	Intermediate	Brown	
FKZ 21-001	0	1	0	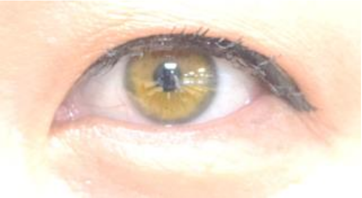
FKZ 21-003	0	0	1	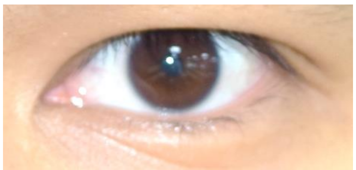
FKZ 21-004	0	0	1	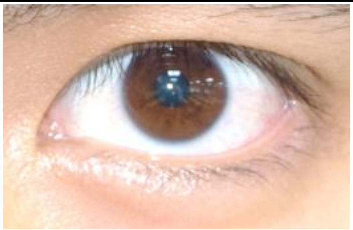
FKZ 21-005	0	0	1	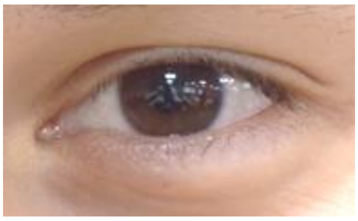
FKZ 21-006	0	0	1	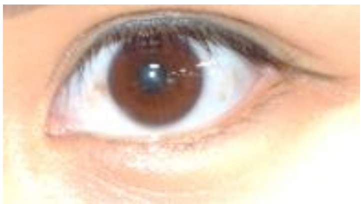
FKZ 21-007	0	0	1	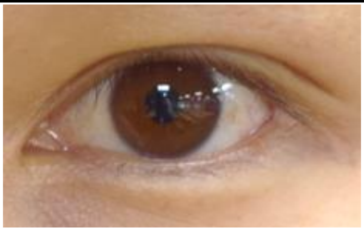
FKZ 21-008	0	0	1	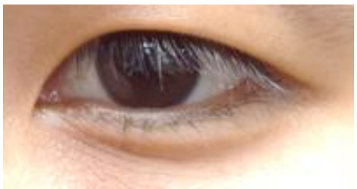

FKZ 21-009	0	0	1	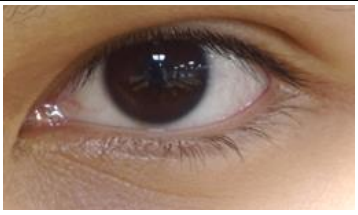
FKZ 21-010	0	0	1	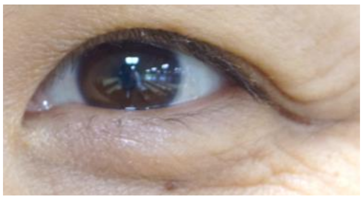
FKZ 21-011	0	0	1	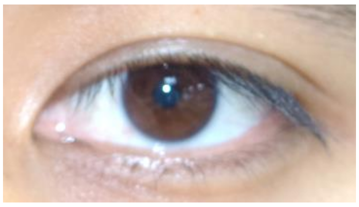
FKZ 21-012	0	1	0	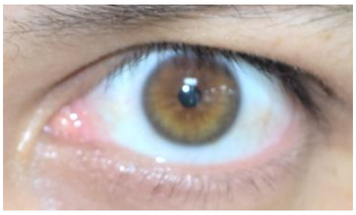
FKZ 21-013	0	1	0	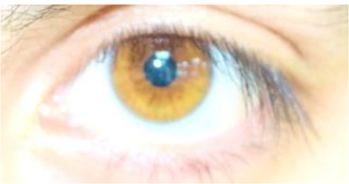
FKZ 21-014	0	0	1	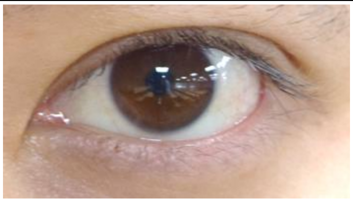
FKZ 21-015	0	0	1	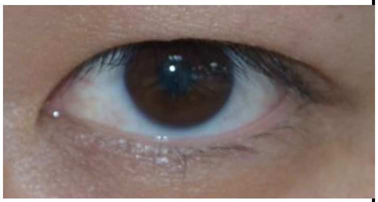
FKZ 21-016	0	0	1	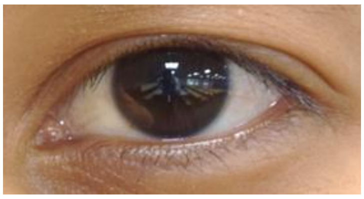

FKZ 21-017	0	1	0	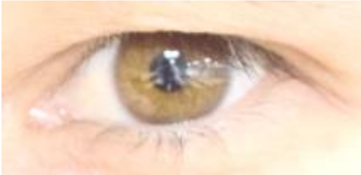
FKZ 21-018	0	0	1	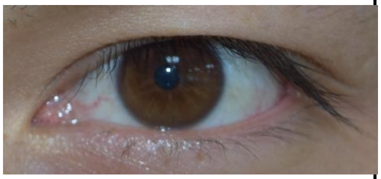
FKZ 21-019	0	0	1	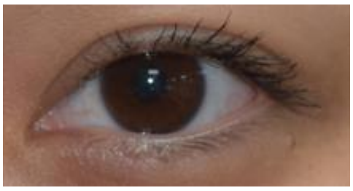
FKZ 21-020	0	0	1	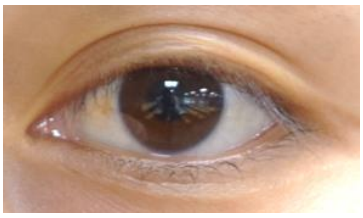
FKZ 21-021	1	0	0	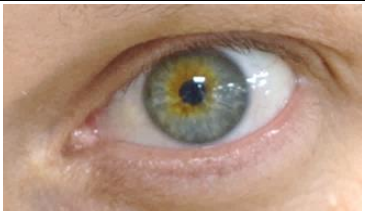
FKZ 21-022	0	0	1	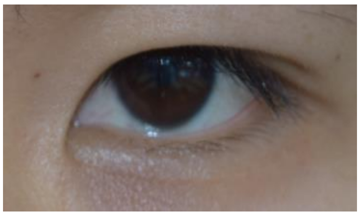
FKZ 21-023	0	0	1	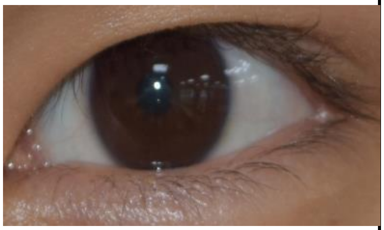
FKZ 21-025	0	0	1	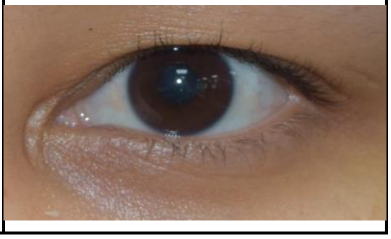

FKZ 21-027	0	0	1	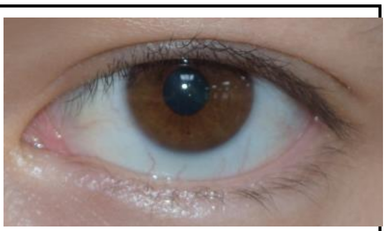
FKZ 21-028	0	0	1	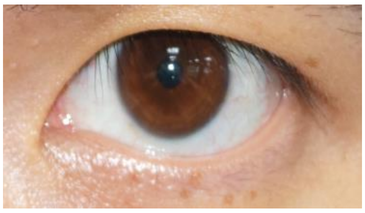
FKZ 21-029	0	1	0	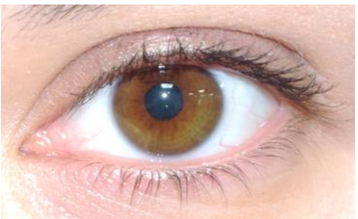
FKZ 21-032	0	0	1	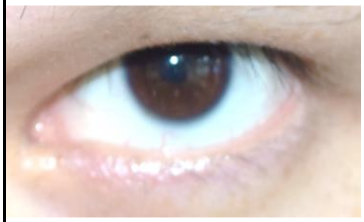
FKZ 21-034	0	0	1	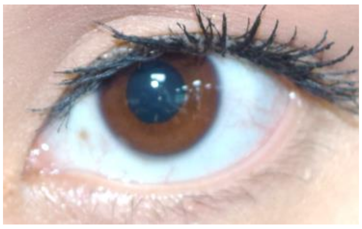
FKZ 21-035	0	0	1	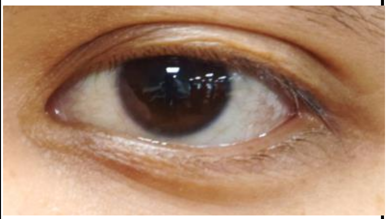
FKZ 21-037	0	1	0	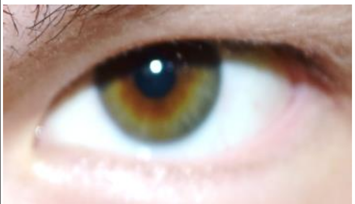
FKZ 21-038	0	1	0	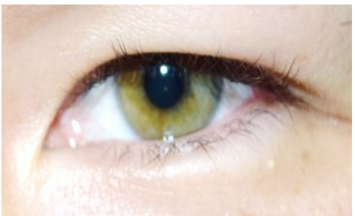

FKZ 21-041	0	0	1	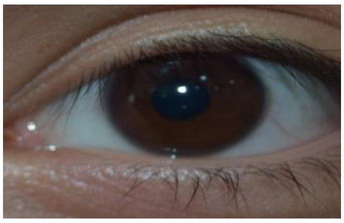
FKZ 21-042	0	0	1	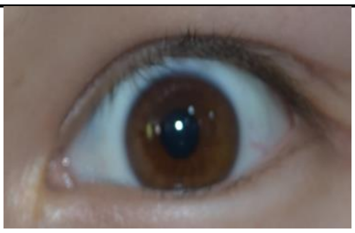
FKZ 21-043	0	0	1	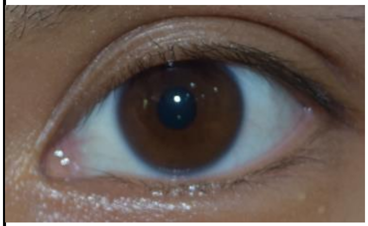
FKZ 21-044	0	0	1	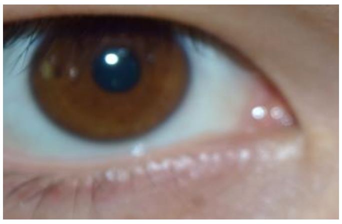
FKZ 21-045	0	1	0	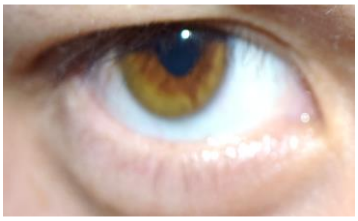
FKZ 21-046	0	0	1	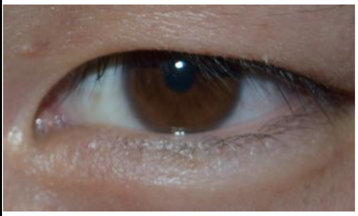
FKZ 21-047	0	0	1	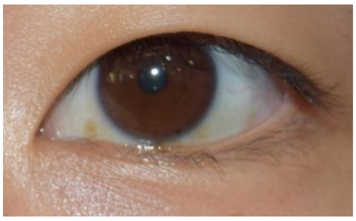
FKZ 21-048	0	0	1	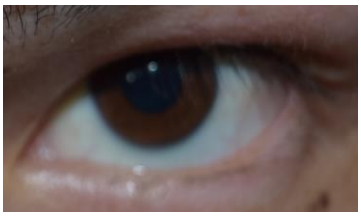

FKZ 21-050	0	0	1	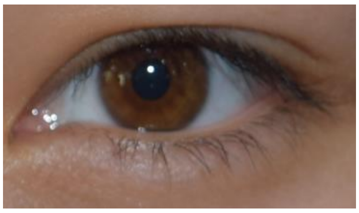
FKZ 21-051	0	0	1	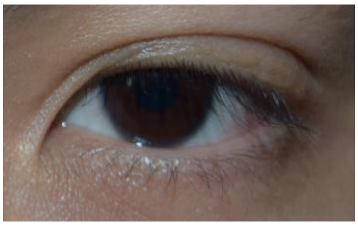
FKZ 21-052	0	0	1	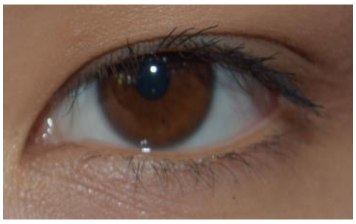
FKZ 21-053	0	0	1	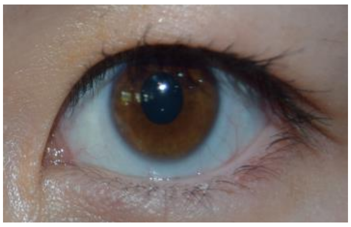
FKZ 21-054	0	0	1	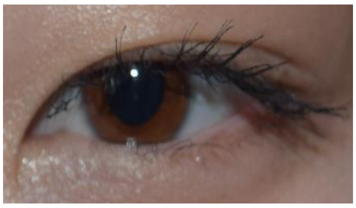
FKZ 21-056	0	0	1	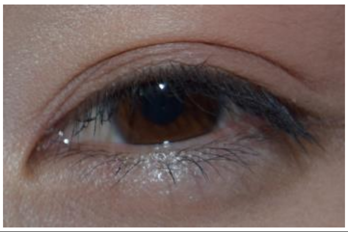 3
FKZ 21-058	0	0	1	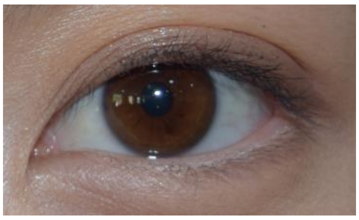
FKZ 21-059	0	0	1	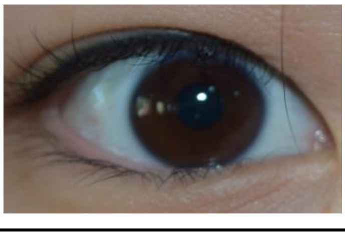

FKZ 21-061	0	0	1	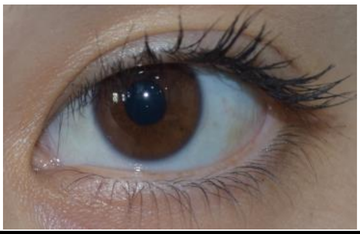
FKZ 21-063	0	0	1	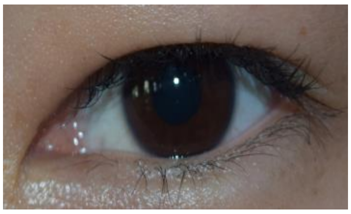
FKZ 21-064	0	0	1	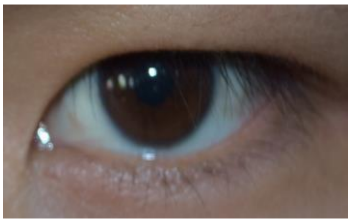
FKZ 21-065	0	0	1	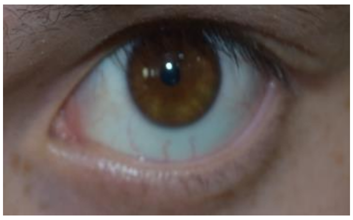
FKZ 21-066	0	0	1	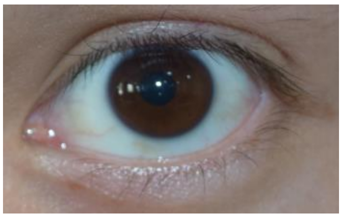
FKZ 21-067	0	0	1	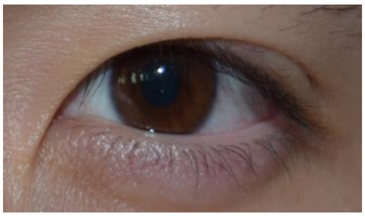
FKZ 21-068	0	0	1	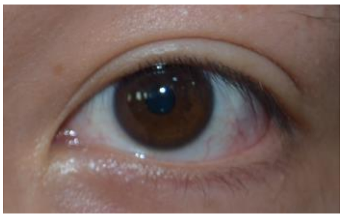
FKZ 21-069	0	0	1	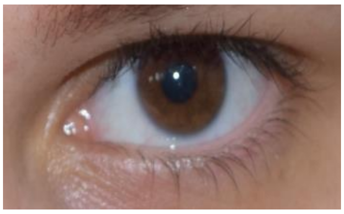

FKZ 21-070	0	0	1	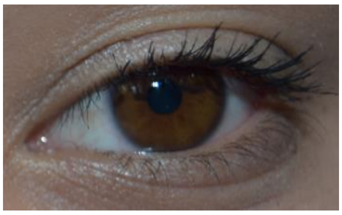
FKZ 21-071	0	1	0	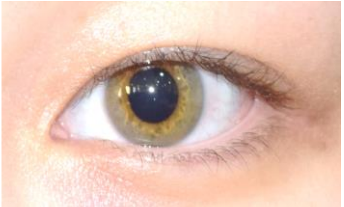
FKZ 21-072	0	0	1	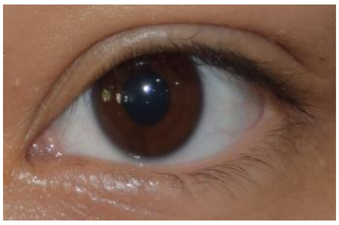
FKZ 21-074	0	0	1	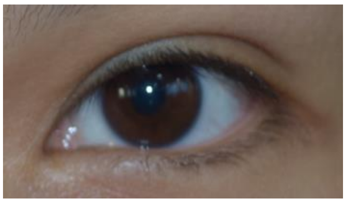
FKZ 21-075	0	0	1	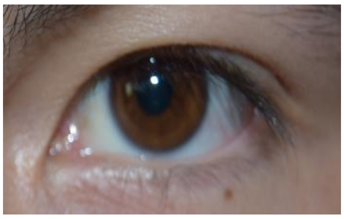
FKZ 21-076	0	0	1	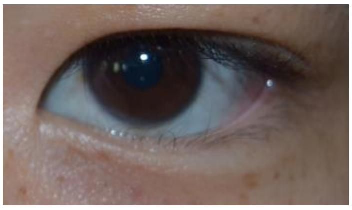
FKZ 21-078	0	0	1	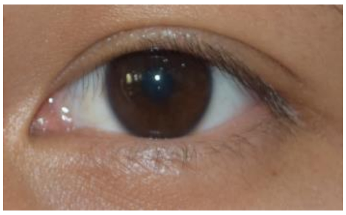
FKZ 21-079	0	0	1	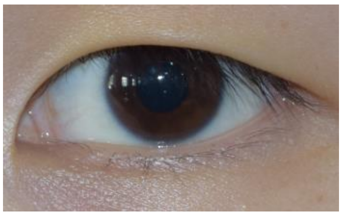

FKZ 21-081	0	0	1	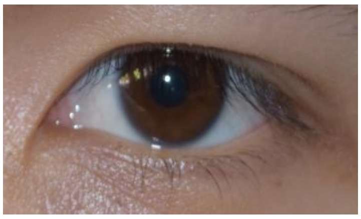
FKZ 21-082	0	0	1	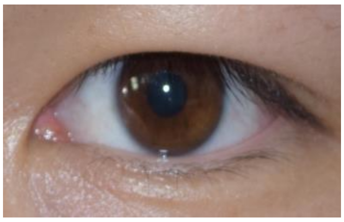
FKZ 21-083	0	0	1	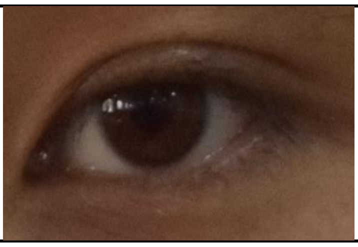
FKZ 21-084	0	0	1	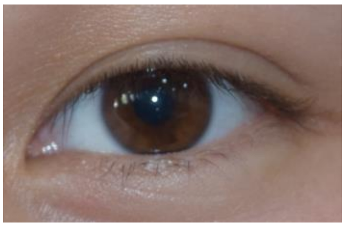
FKZ 21-085	0	1	0	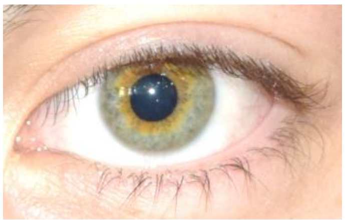
FKZ 21-086	0	0	1	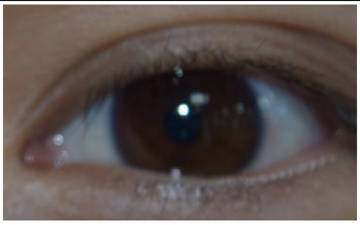
FKZ 21-087	0	0	1	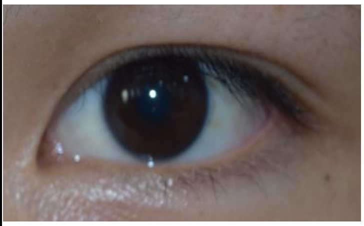
FKZ 21-088	0	0	1	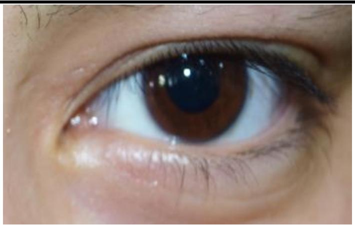

FKZ 21-091	0	1	0	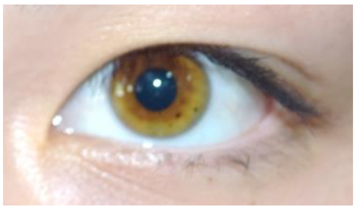
FKZ 21-092	0	1	0	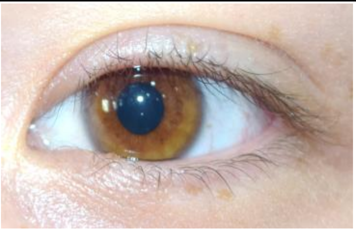
FKZ 21-093	0	0	1	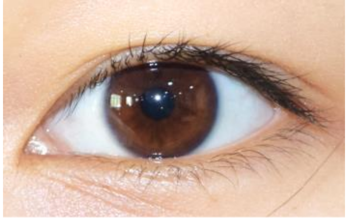
FKZ 21-094	0	0	1	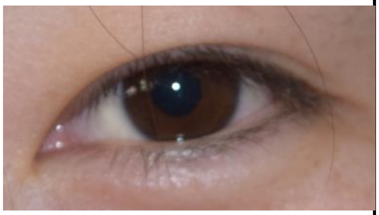
FKZ 21-097	0	0	1	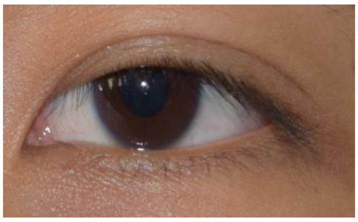
FKZ 21-098	0	0	1	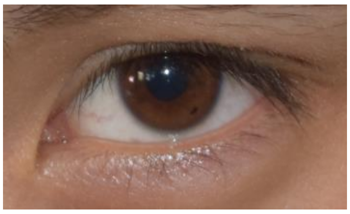
FKZ 21-100	0	0	1	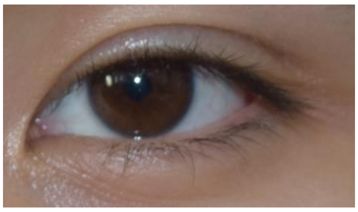
FKZ 21-101	0	0	1	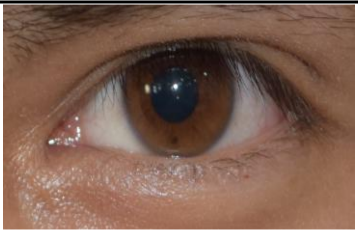

FKZ 21-102	0	0	1	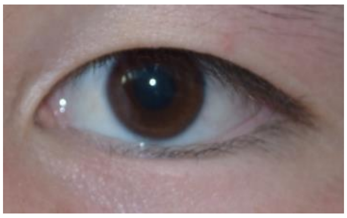
FKZ 21-105	0	1	0	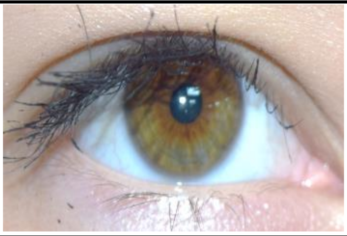
FKZ 21-106	0	1	0	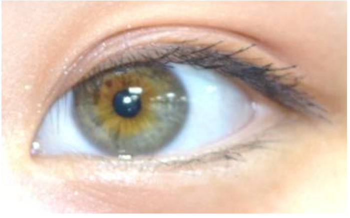
FKZ 21-107	0	0	1	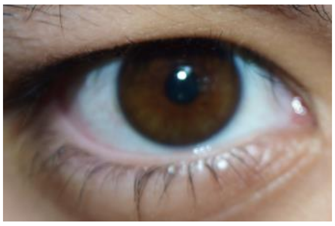
FKZ 21-108	0	0	1	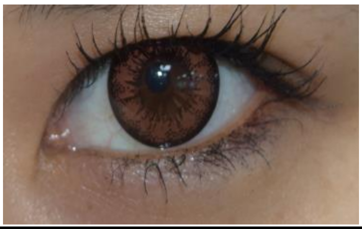
FKZ 21-109	0	0	1	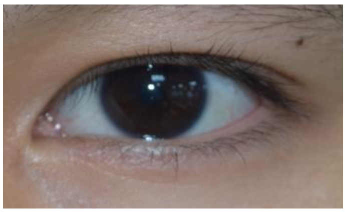
FKZ 21-110	0	0	1	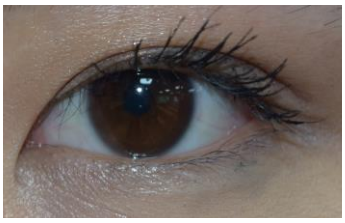
FKZ 21-111	0	0	1	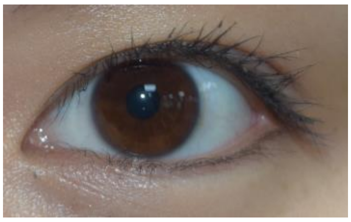

FKZ 21-114	0	0	1	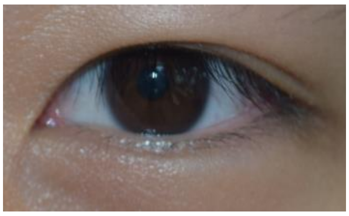
FKZ 21-116	0	0	1	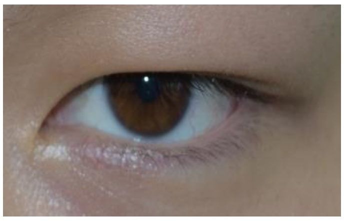
FKZ 21-117	0	0	1	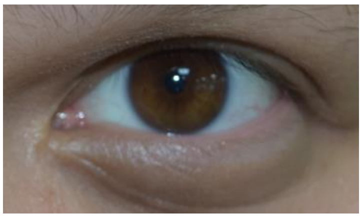
FKZ 21-118	0	1	0	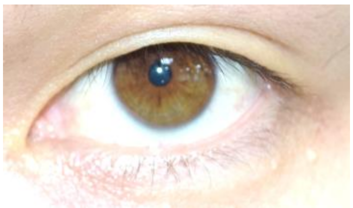
FKZ 21-119	0	0	1	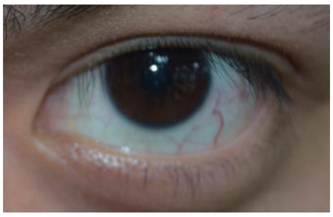
FKZ 21-120	0	0	1	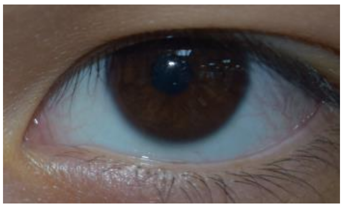
FKZ 21-122	0	0	1	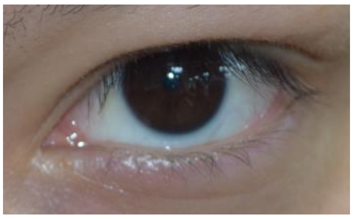
FKZ 21-123	0	1	0	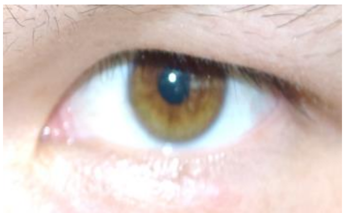

FKZ 21-124	0	0	1	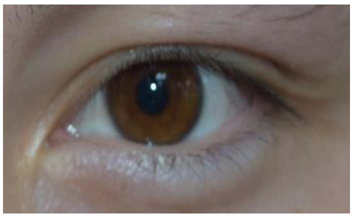
FKZ 21-125	0	0	1	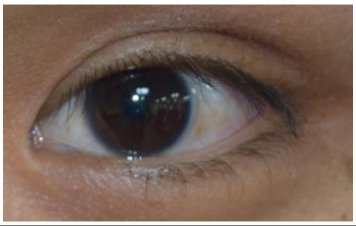
FKZ 21-126	0	0	1	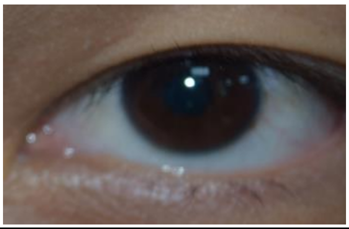
FKZ 21-127	0	0	1	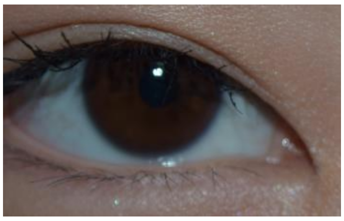
FKZ 21-128	0	0	1	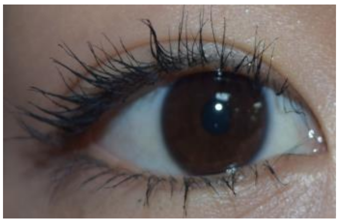
FKZ 21-129	0	0	1	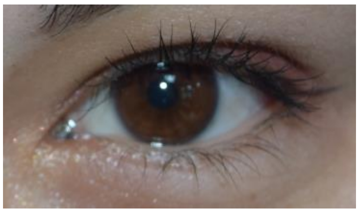
FKZ 21-130	0	0	1	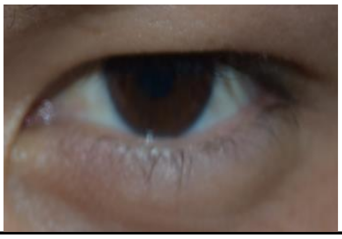
FKZ 21-131	0	0	1	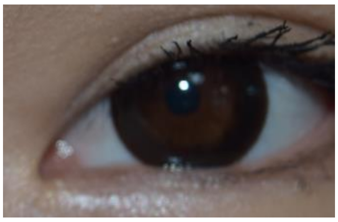

FKZ 21-132	0	0	1	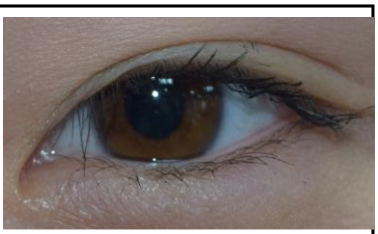
FKZ 21-133	0	0	1	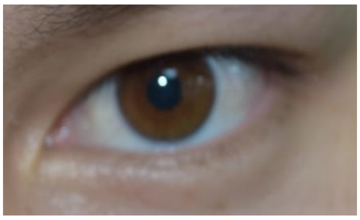
FKZ 21-134	0	1	0	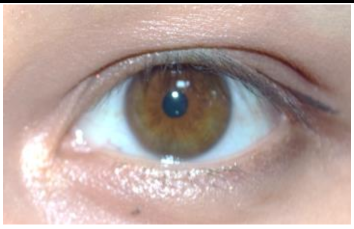
FKZ 21-136	0	0	1	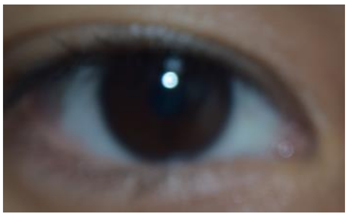
FKZ 21-138	0	0	1	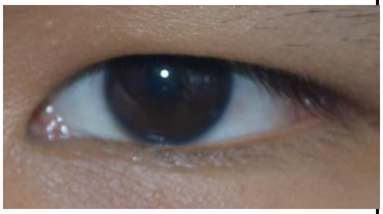
FKZ 21-139	0	0	1	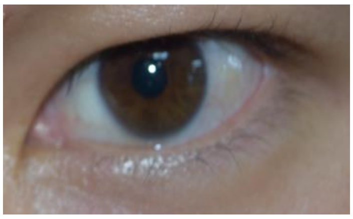
FKZ 21-140	0	0	1	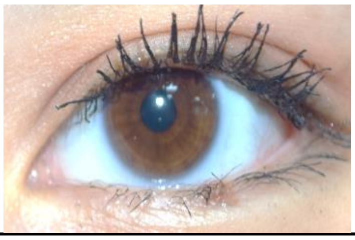
FKZ 21-141	0	0	1	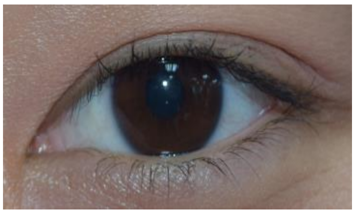

FKZ 21-142	0	0	1	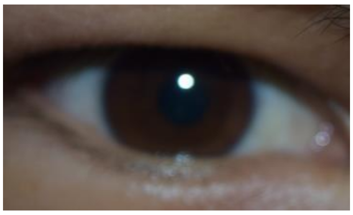
FKZ 21-143	0	1	0	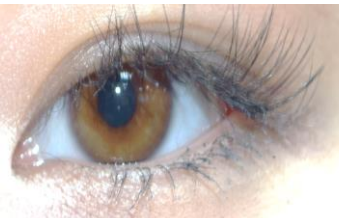
FKZ 21-144	0	0	1	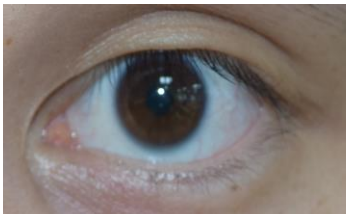
FKZ 21-148	0	0	1	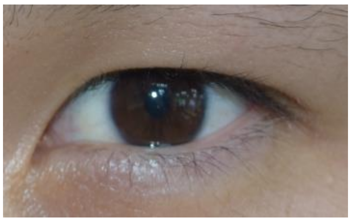
FKZ 21-149	0	0	1	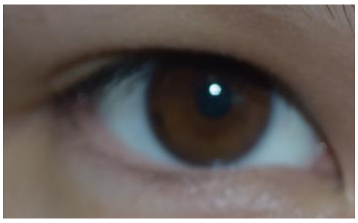
FKZ 21-150	0	1	0	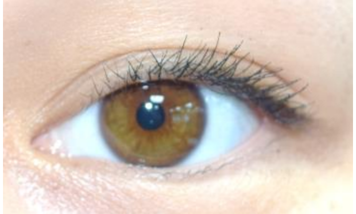
FKZ 21-151	0	0	1	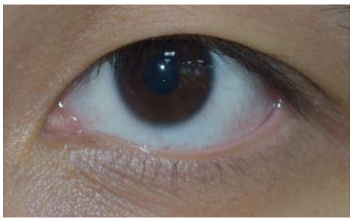
FKZ 21-152	0	0	1	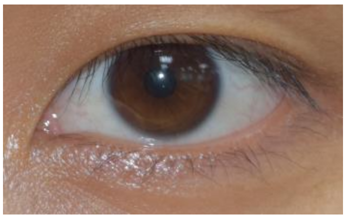

FKZ 21-153	0	0	1	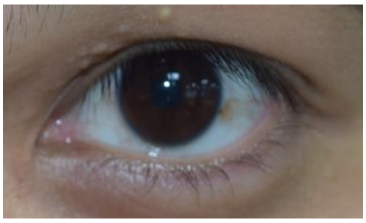
FKZ 21-154	0	1	0	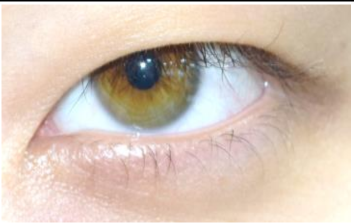
FKZ 21-155	0	0	1	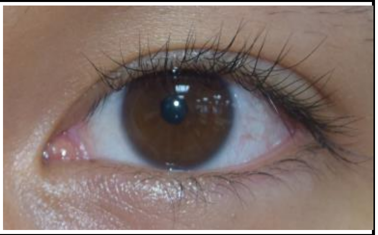
FKZ 21-156	0	0	1	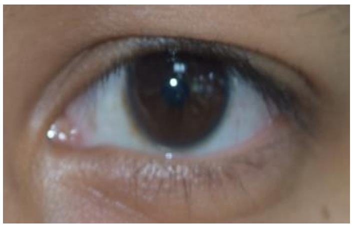
FKZ 21-157	0	0	1	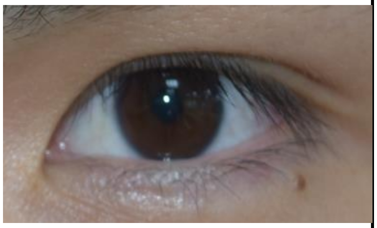
FKZ 21-158	0	1	0	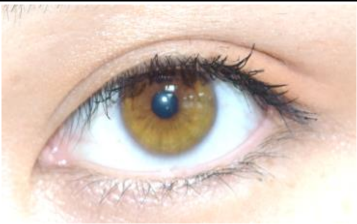
FKZ 21-159	0	0	1	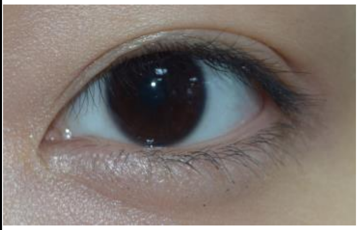
FKZ 21-160	0	0	1	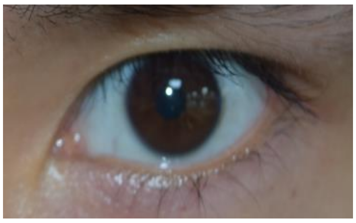

FKZ 21-161	0	0	1	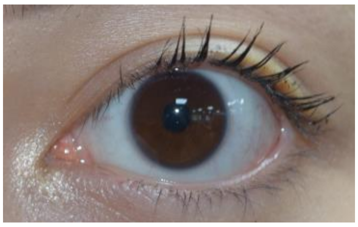
FKZ 21-162	0	1	0	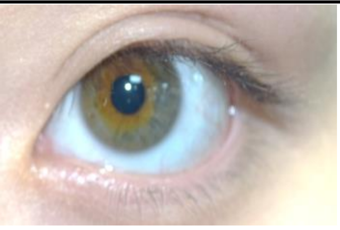
FKZ 21-163	0	0	1	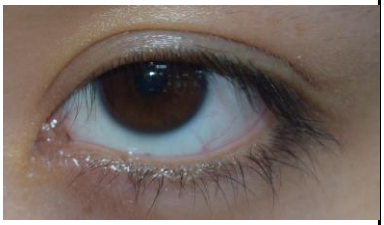
FKZ 21-164	0	0	1	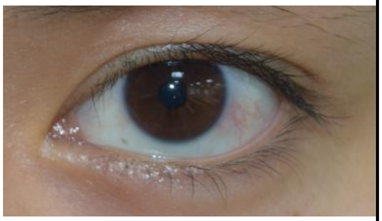
FKZ 21-167	0	0	1	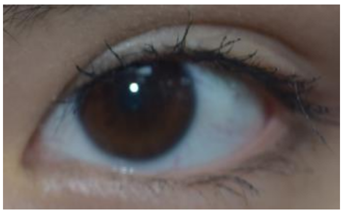
FKZ 21-168	0	1	0	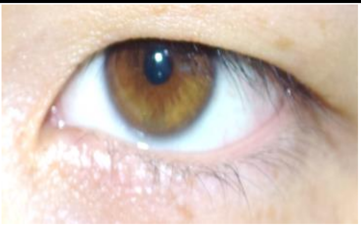
FKZ 21-169	0	0	1	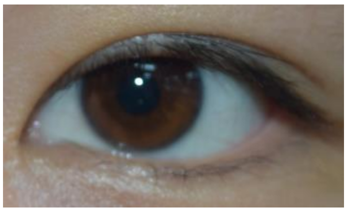
FKZ 21-170	0	0	1	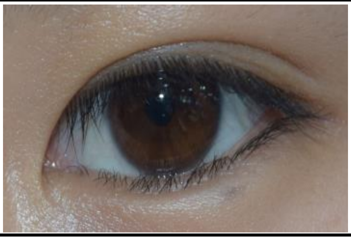

FKZ 21-171	0	0	1	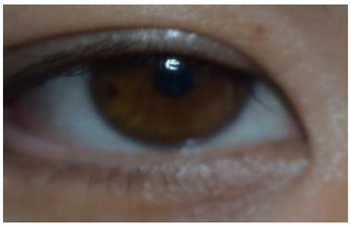
FKZ 21-172	0	0	1	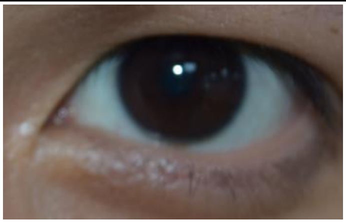
FKZ 21-173	0	0	1	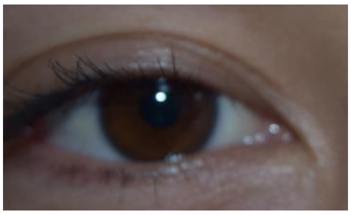
FKZ 21-174	0	0	1	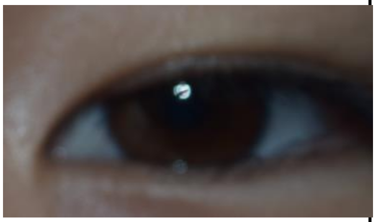
FKZ 21-175	0	0	1	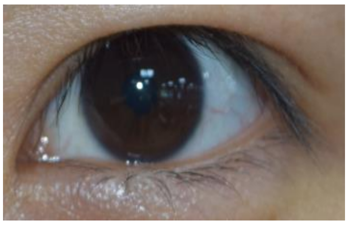
FKZ 21-176	0	0	1	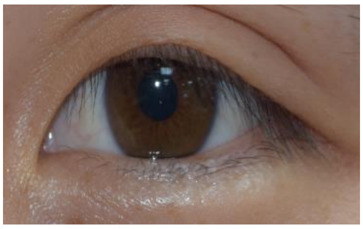
FKZ 21-177	0	0	1	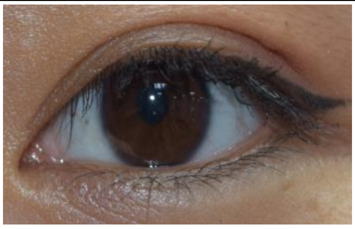
FKZ 21-178	0	0	1	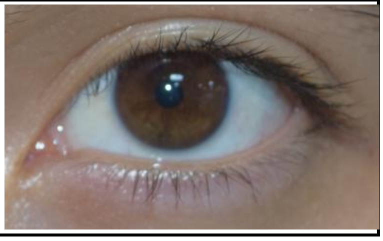

FKZ 21-179	0	0	1	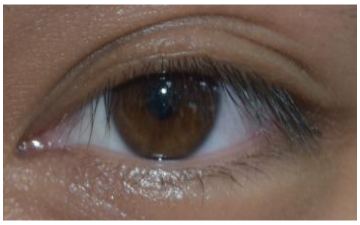
FKZ 21-180	0	0	1	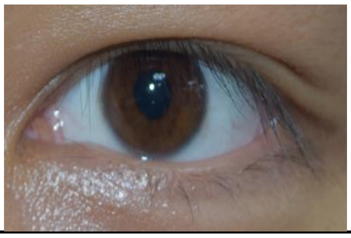
FKZ 21-181	0	1	0	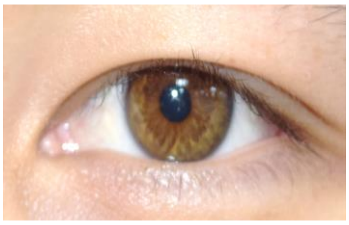
FKZ 21-182	0	0	1	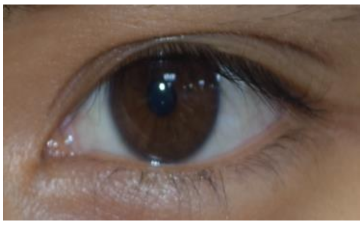
FKZ 21-183	0	0	1	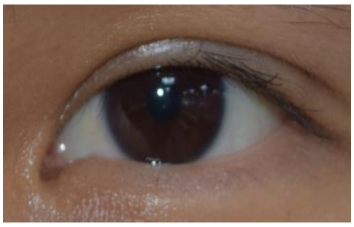
FKZ 21-184	0	0	1	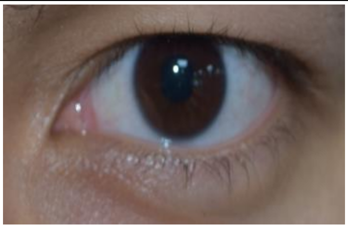
FKZ 21-185	0	0	1	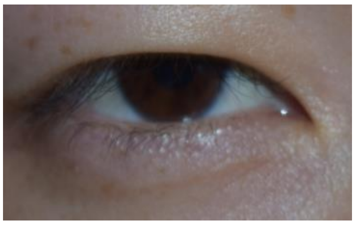
FKZ 21-186	0	0	1	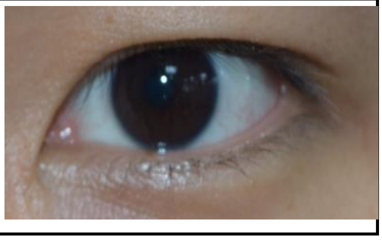

FKZ 21-187	0	0	1	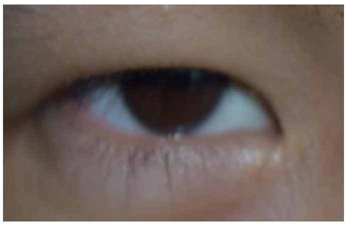
FKZ 21-188	0	1	0	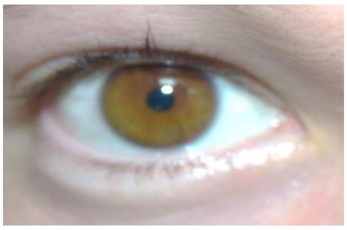
FKZ 21-189	0	0	1	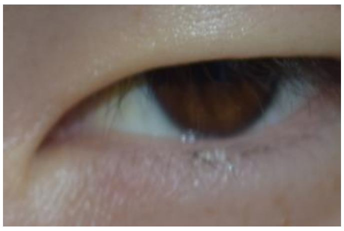
FKZ 21-190	0	0	1	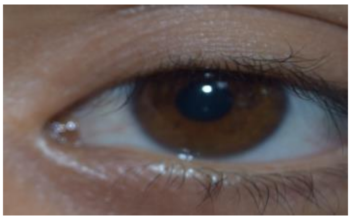
FKZ 21-191	0	0	1	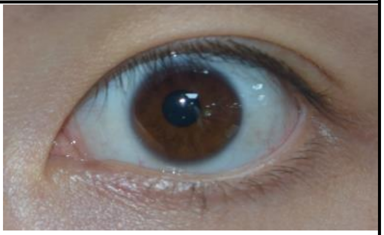
FKZ 21-192	0	0	1	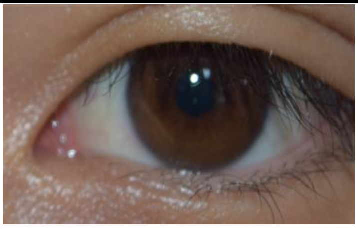
FKZ 21-193	0	1	0	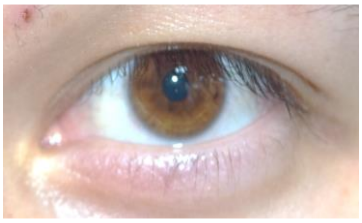
FKZ 21-194	0	0	1	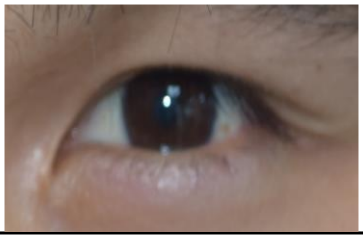

FKZ 21-195	0	0	1	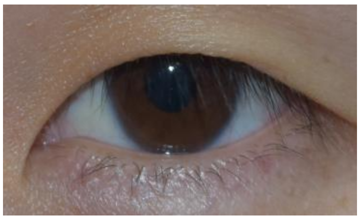
FKZ 21-196	0	0	1	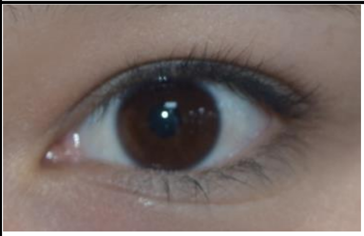
FKZ 21-197	0	0	1	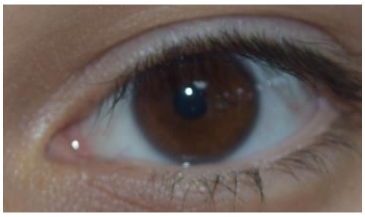
FKZ 21-198	0	0	1	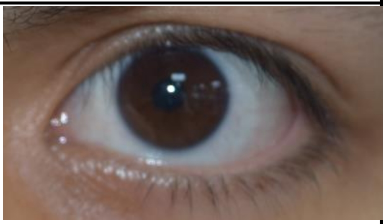
FKZ 21-199	0	0	1	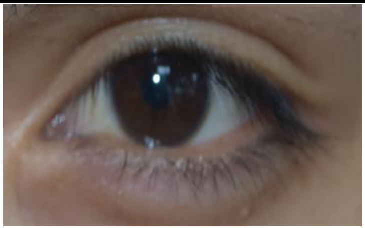
FKZ 21-200	0	0	1	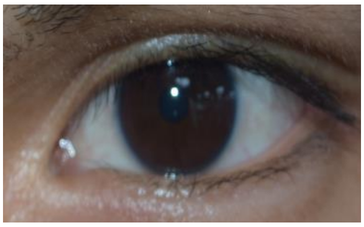
FKZ 21-201	0	0	1	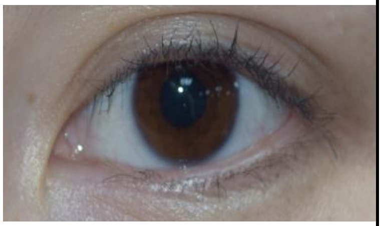
FKZ 21-203	0	0	1	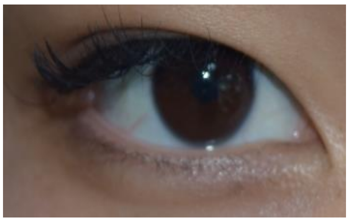

FKZ 21-204	0	0	1	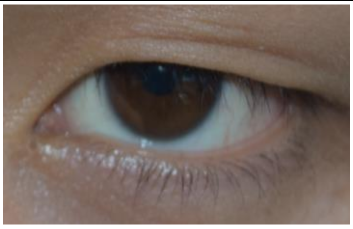
FKZ 21-205	0	0	1	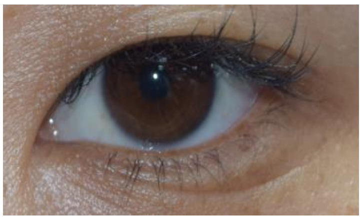
FKZ 21-206	0	0	1	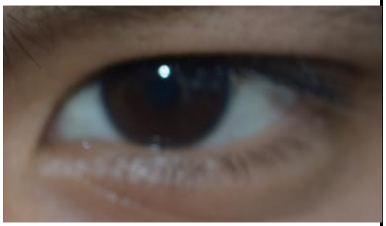
FKZ 21-207	0	0	1	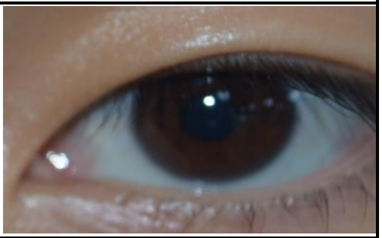
FKZ 21-208	0	0	1	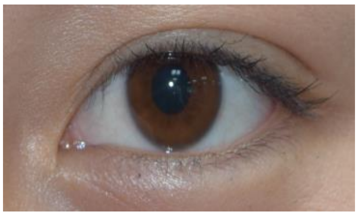
FKZ 21-210	0	1	0	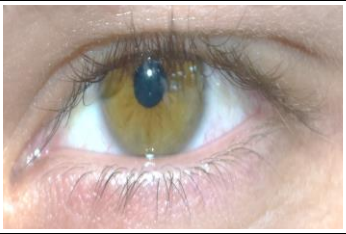
FKZ 21-211	0	0	1	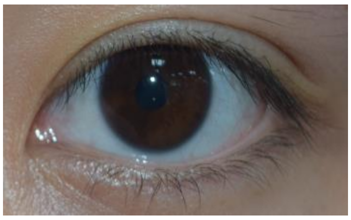
FKZ 21-212	0	0	1	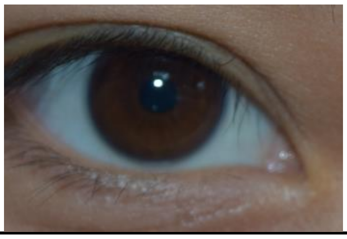

FKZ 21-213	0	0	1	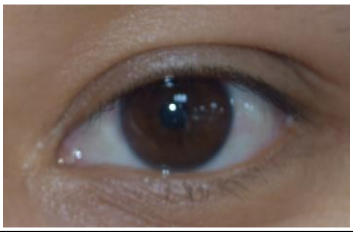
FKZ 21-214	0	0	1	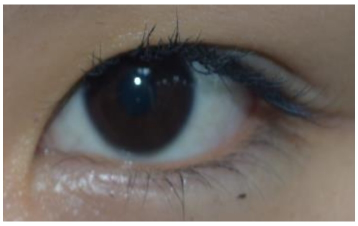
FKZ 21-215	0	0	1	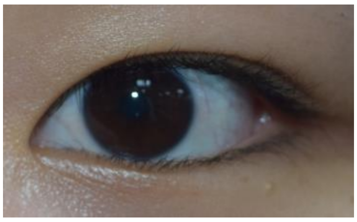
FKZ 21-216	0	1	0	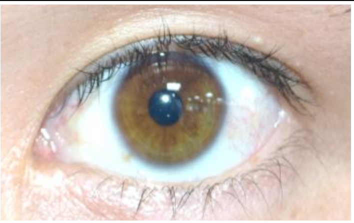
FKZ 21-217	0	0	1	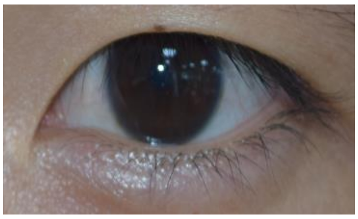
FKZ 21-219	0	0	1	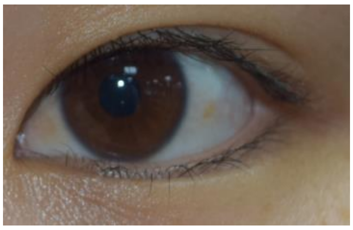
FKZ 21-220	0	0	1	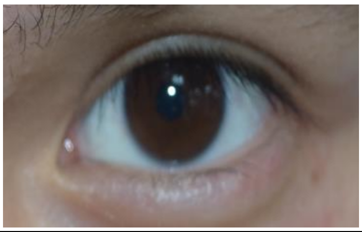
FKZ 21-221	0	0	1	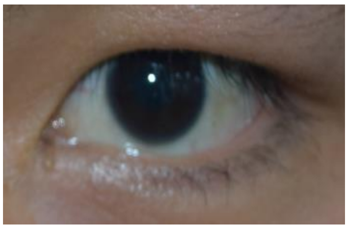

FKZ 21-222	0	0	1	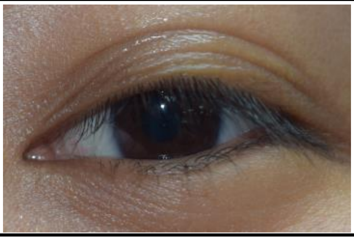
FKZ 21-223	0	0	1	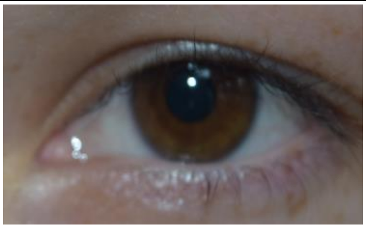
FKZ 21-225	0	0	1	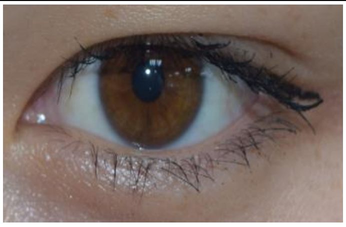
FKZ 21-226	0	0	1	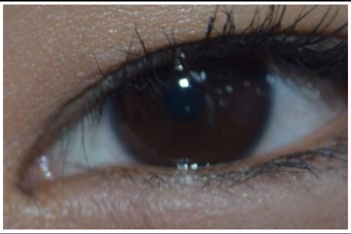
FKZ 21-227	0	0	1	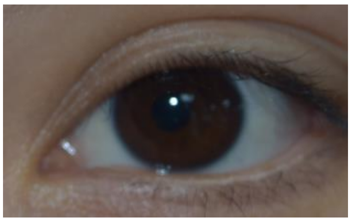
FKZ 21-228	0	0	1	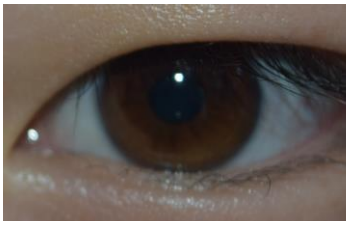
FKZ 21-229	0	0	1	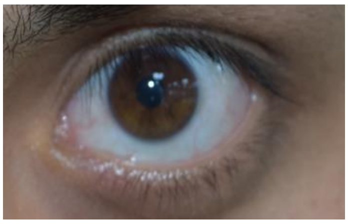
FKZ 21-230	0	0	1	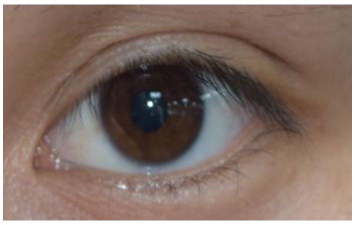

FKZ 21-231	0	0	1	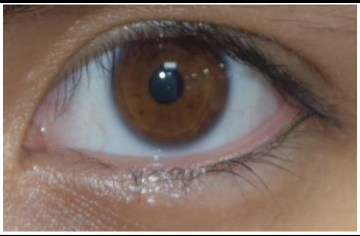
FKZ 21-232	0	0	1	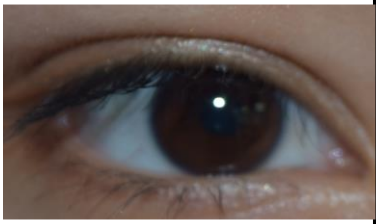
FKZ 21-233	0	1	0	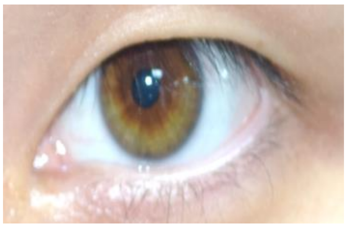
FKZ 21-234	0	0	1	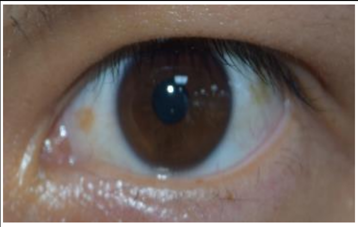
FKZ 21-235	0	0	1	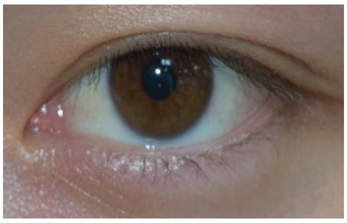
FKZ 21-236	0	1	0	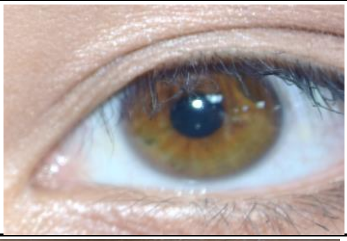
FKZ 21-237	0	0	1	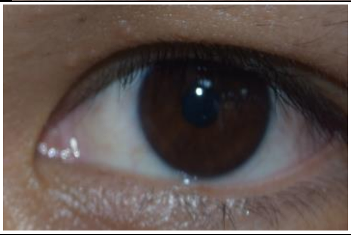
FKZ 21-238	0	0	1	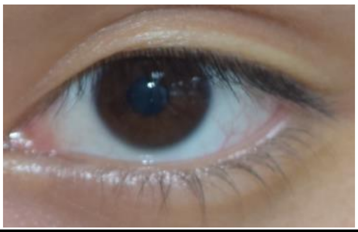

FKZ 21-239	0	0	1	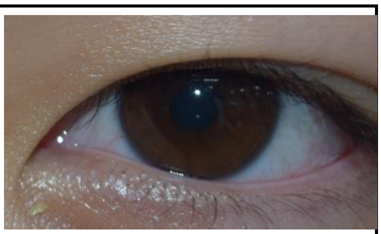
FKZ 21-240	0	0	1	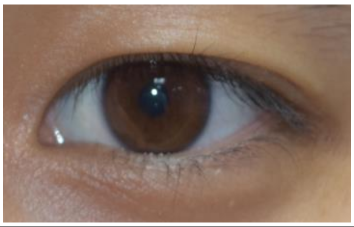
FKZ 21-241	1	0	0	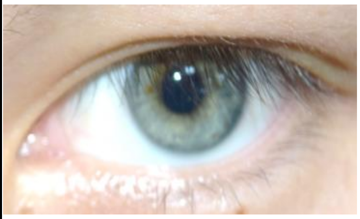
FKZ 21-242	0	0	1	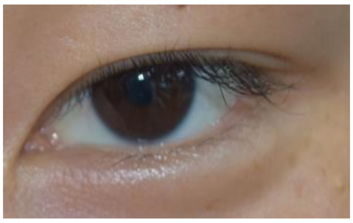
FKZ 21-244	0	0	1	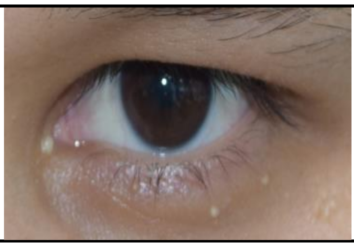
FKZ 21-245	0	0	1	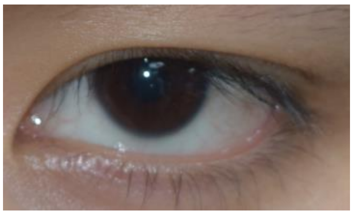
FKZ 21-246	0	0	1	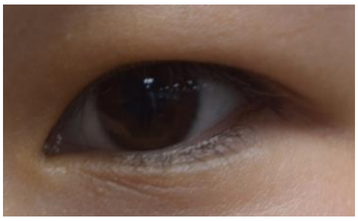
FKZ 21-247	0	0	1	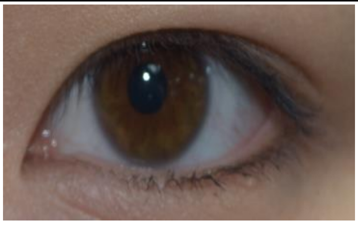

FKZ 21-248	0	0	1	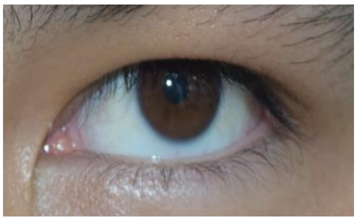
FKZ 21-249	0	0	1	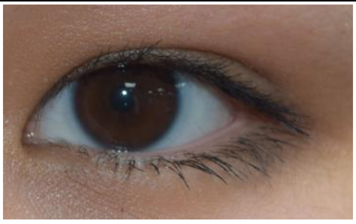
FKZ 21-250	0	1	0	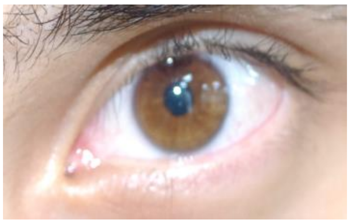
FKZ 21-251	0	0	1	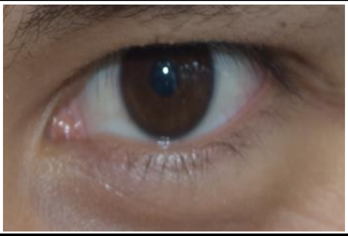
FKZ 21-252	0	0	1	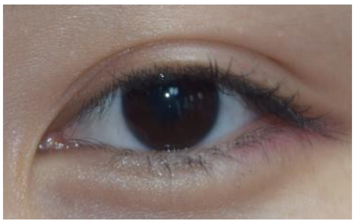
FKZ 21-254	0	0	1	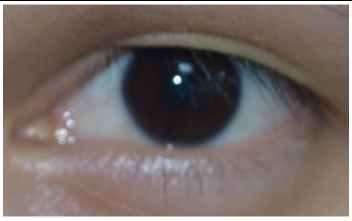
FKZ 21-256	0	0	1	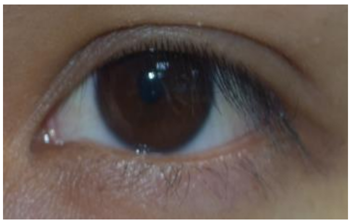
FKZ 21-257	0	0	1	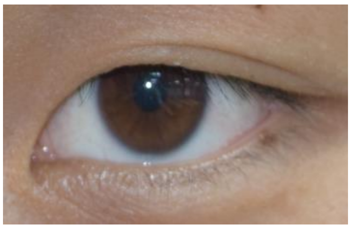

FKZ 21-258	0	0	1	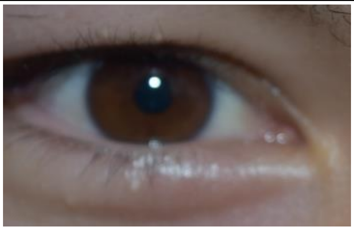
FKZ 21-259	0	0	1	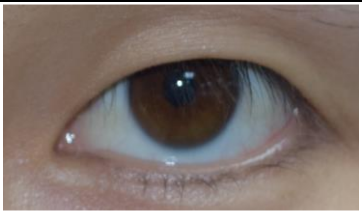
FKZ 21-260	0	0	1	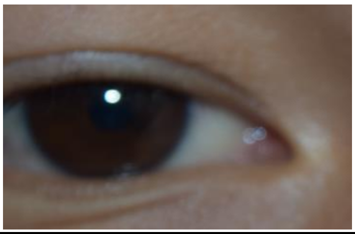
FKZ 21-261	0	0	1	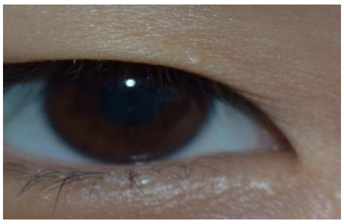
FKZ 21-262	0	1	0	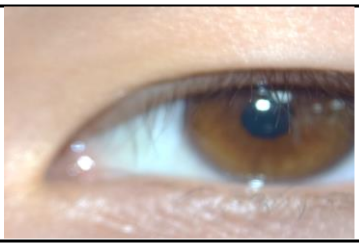
FKZ 21-264	0	0	1	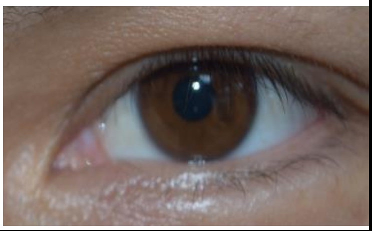
FKZ 21-265	0	0	1	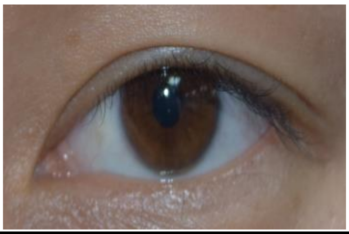
FKZ 21-266	0	0	1	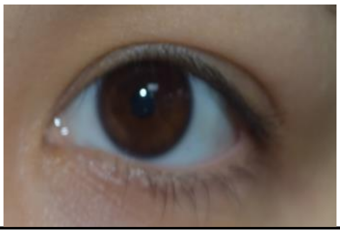

FKZ 21-267	0	0	1	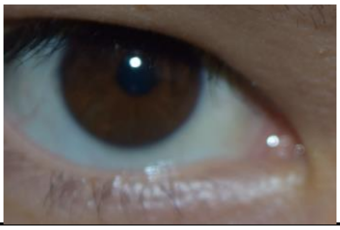
FKZ 21-268	0	0	1	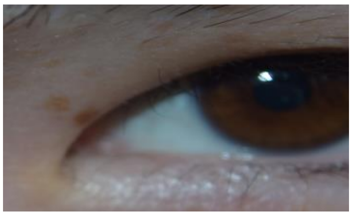
FKZ 21-269	0	0	1	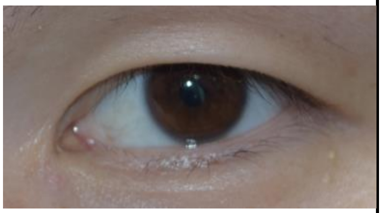
FKZ 21-270	0	0	1	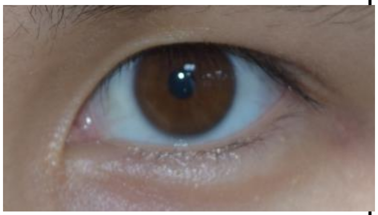
FKZ 21-271	0	0	1	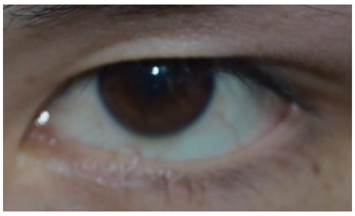
FKZ 21-272	0	0	1	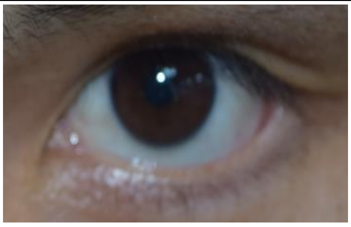
FKZ 21-273	0	1	0	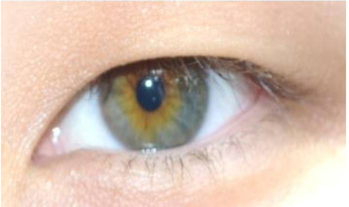
FKZ 21-274	0	0	1	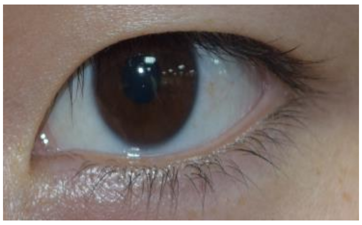

FKZ 21-275	0	0	1	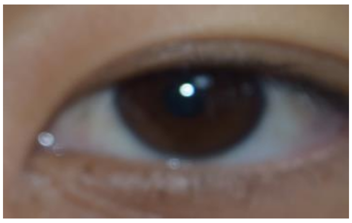
FKZ 21-276	0	0	1	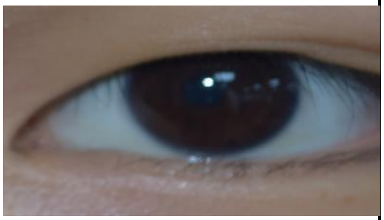
FKZ 21-278	0	0	1	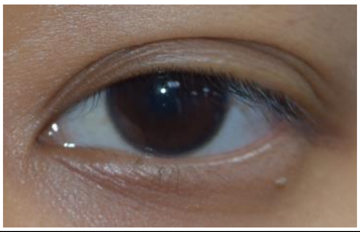
FKZ 21-279	0	0	1	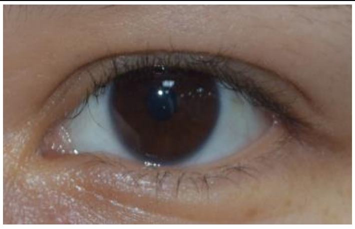
FKZ 21-280	0	0	1	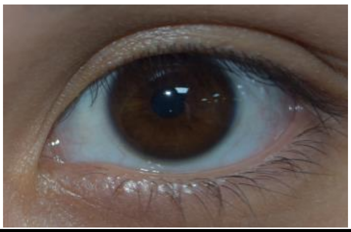
FKZ 21-281	0	1	0	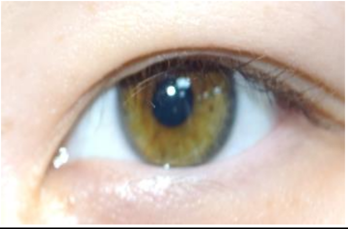
FKZ 21-282	0	0	1	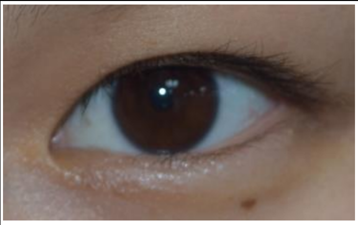
FKZ 21-283	0	0	1	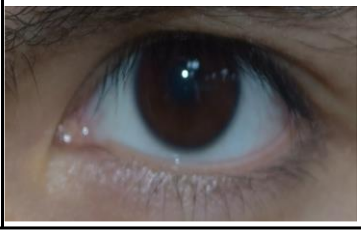

FKZ 21-284	0	0	1	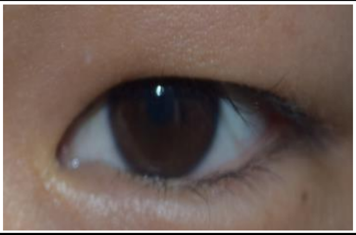
FKZ 21-285	0	0	1	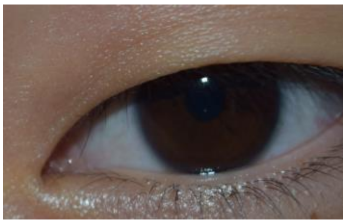
FKZ 21-286	0	1	0	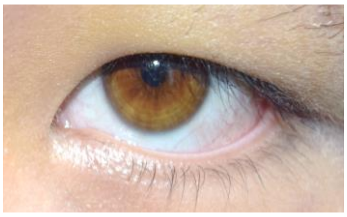
FKZ 21-289	0	0	1	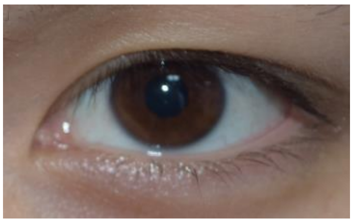
FKZ 21-290	0	0	1	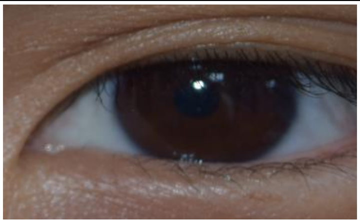
FKZ 21-291	0	1	0	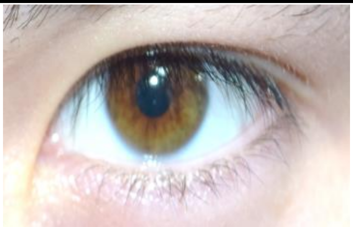
FKZ 21-292	0	0	1	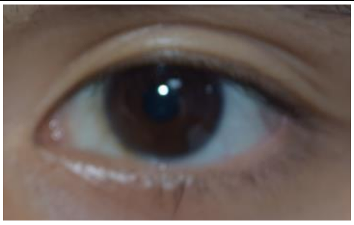
FKZ 21-294	0	0	1	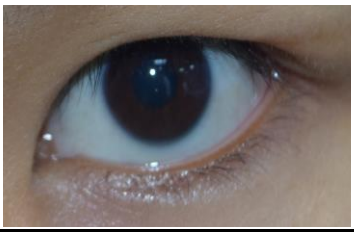

FKZ 21-295	0	0	1	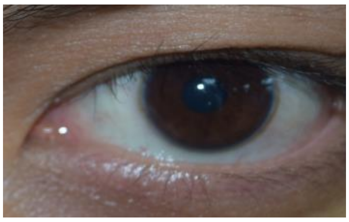
FKZ 21-296	0	0	1	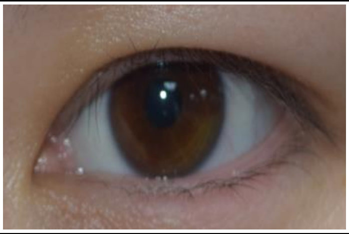
FKZ 21-298	0	1	0	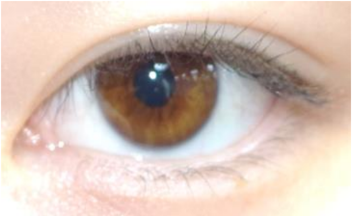
FKZ 21-299	0	1	0	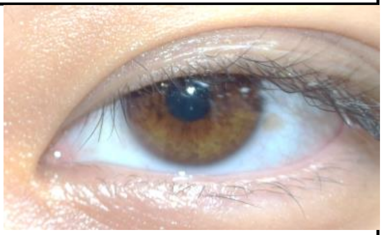
FKZ 21-300	0	0	1	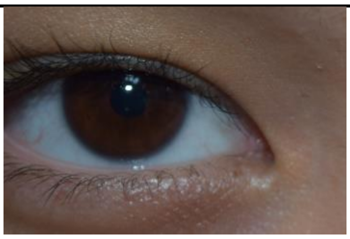
FKZ 21-301	0	0	1	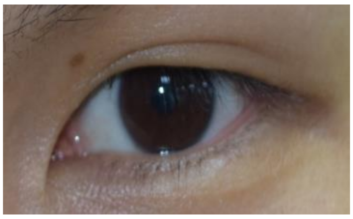
FKZ 21-302	0	0	1	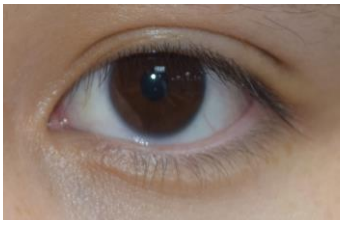
FKZ 21-304	0	0	1	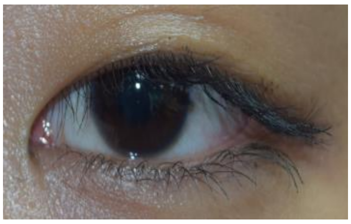

FKZ 21-305	0	0	1	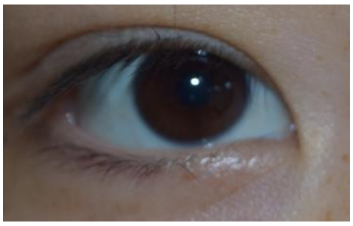
FKZ 21-306	0	0	1	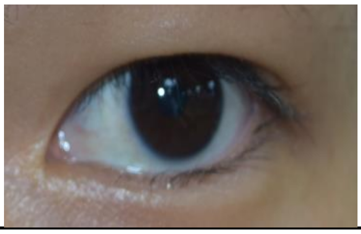
FKZ 21-308	0	0	1	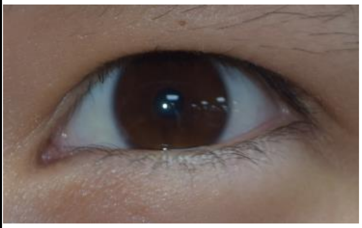
FKZ 21-309	0	0	1	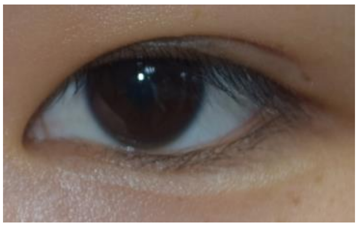
FKZ 21-310	0	0	1	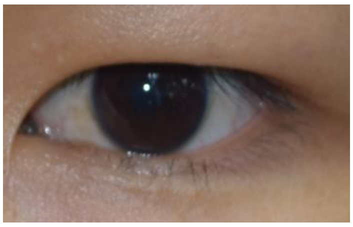
FKZ 21-311	0	0	1	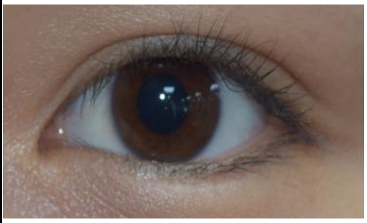
FKZ 21-312	0	0	1	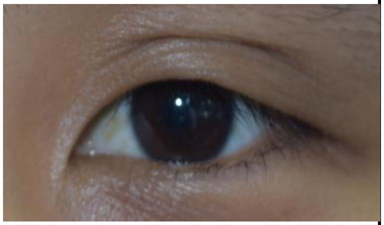
FKZ 21-315	0	0	1	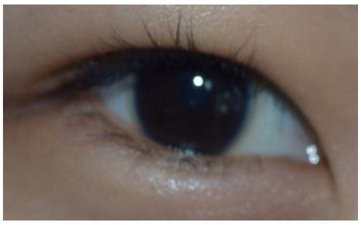

FKZ 21-316	0	0	1	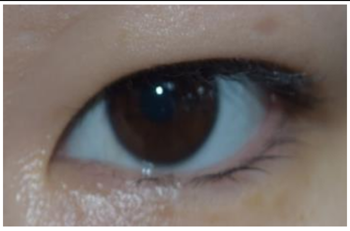
FKZ 21-317	0	1	0	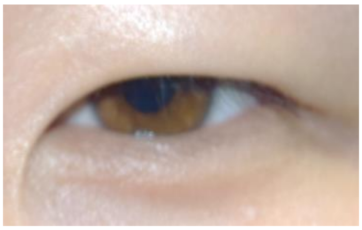
FKZ 21-318	0	0	1	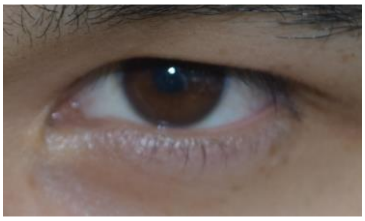
FKZ 21-319	0	0	1	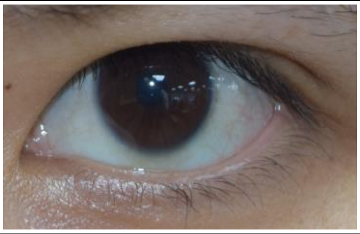
FKZ 21-320	0	0	1	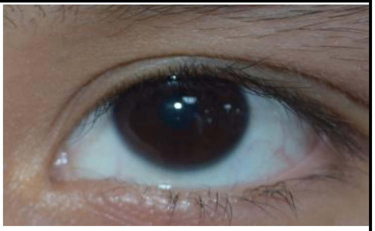
FKZ 21-321	0	0	1	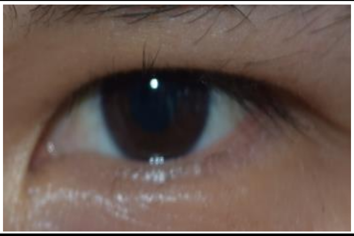
FKZ 21-322	0	0	1	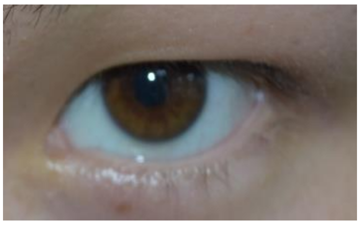
FKZ 21-323	0	0	1	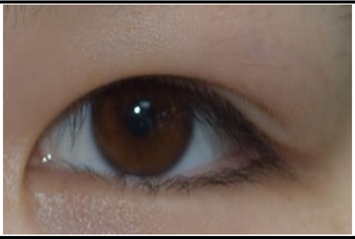

FKZ 21-324	0	0	1	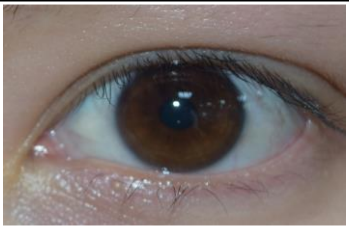
FKZ 21-325	0	0	1	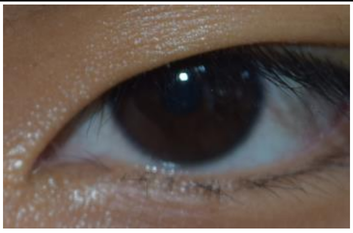
FKZ 21-326	0	0	1	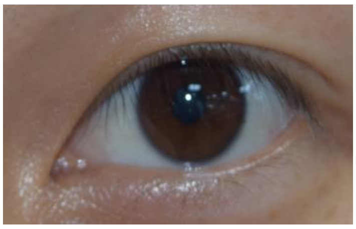
FKZ 21-327	0	0	1	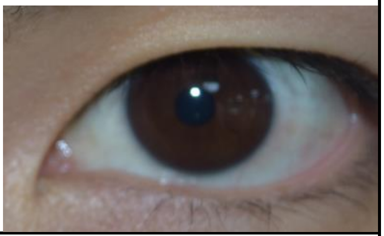
FKZ 21-328	0	0	1	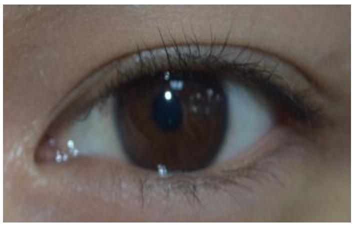
FKZ 21-329	0	0	1	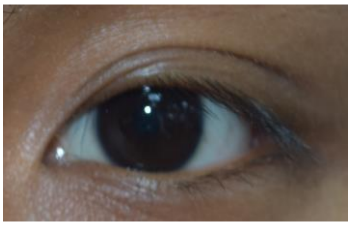
FKZ 21-330	0	0	1	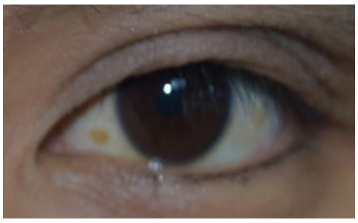
FKZ 21-331	0	0	1	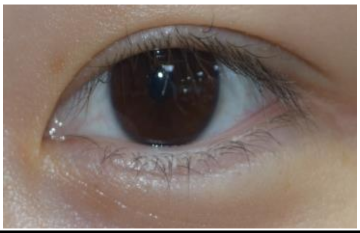

FKZ 21-332	0	1	0	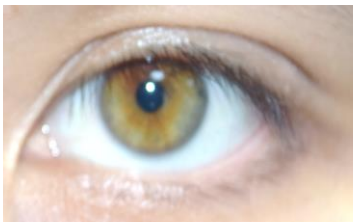
FKZ 21-333	0	0	1	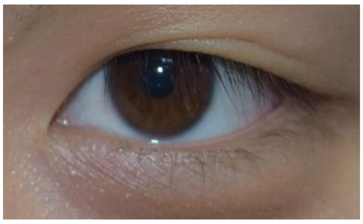
FKZ 21-334	0	0	1	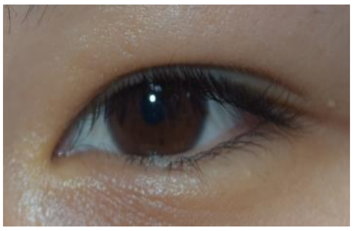
FKZ 21-335	0	0	1	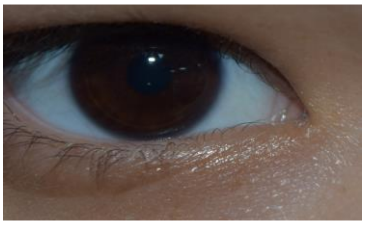
FKZ 21-336	0	0	1	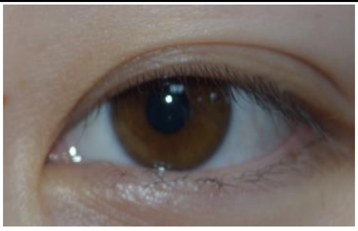
FKZ 21-338	0	0	1	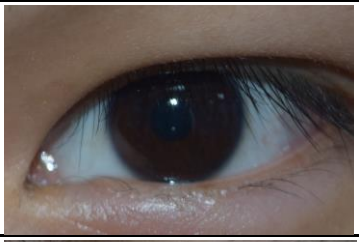
FKZ 21-339	0	0	1	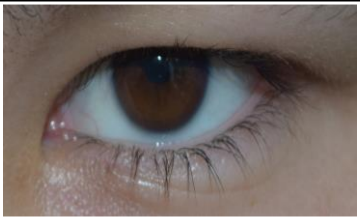
FKZ 21-340	0	0	1	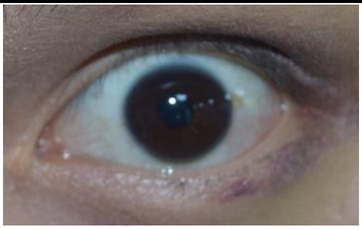

FKZ 21-342	0	0	1	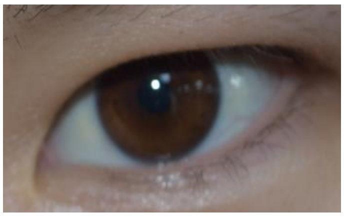
FKZ 21-343	0	0	1	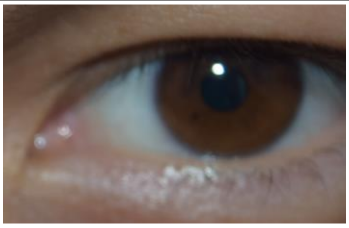
FKZ 21-344	0	0	1	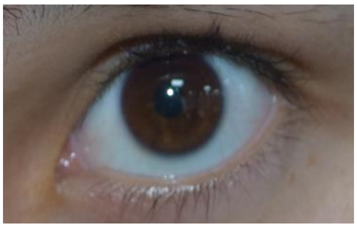
FKZ 21-345	0	0	1	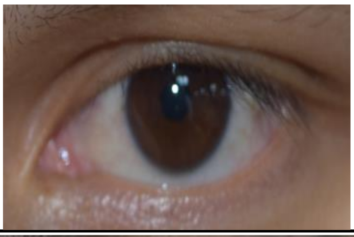
FKZ 21-346	0	0	1	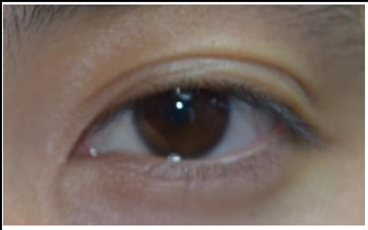
FKZ 21-347	0	0	1	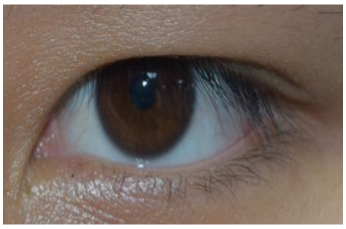
FKZ 21-348	0	0	1	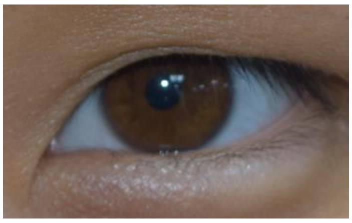
FKZ 21-349	0	0	1	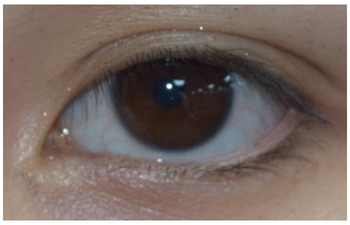

FKZ 21-350	0	0	1	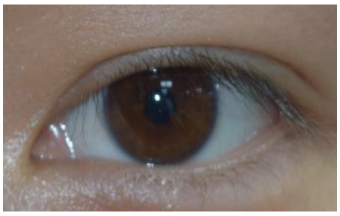
FKZ 21-351	0	0	1	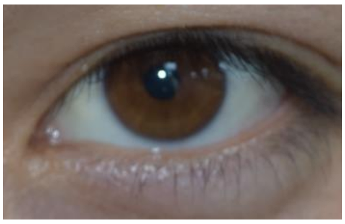
FKZ 21-352	0	0	1	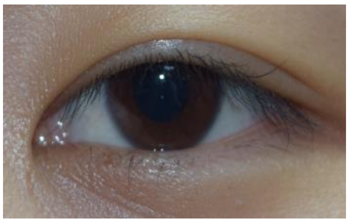
FKZ 21-353	0	0	1	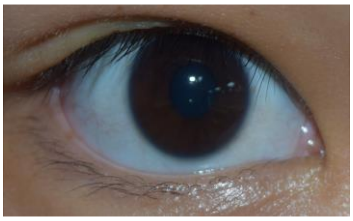
FKZ 21-354	0	0	1	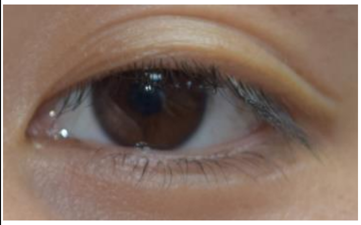
FKZ 21-356	0	0	1	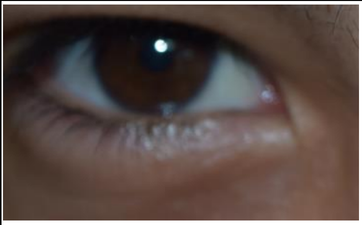
FKZ 21-357	0	1	0	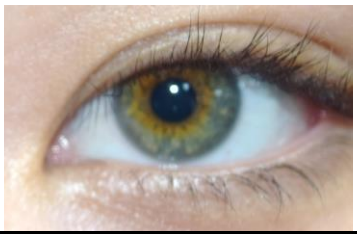
FKZ 21-358	0	0	1	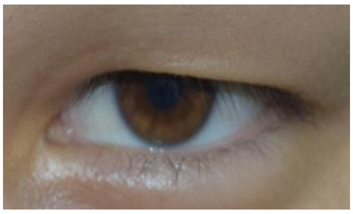

FKZ 21-359	0	0	1	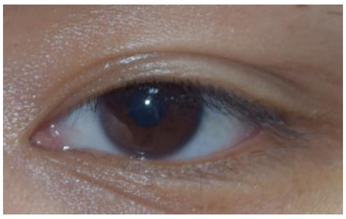
FKZ 21-360	0	0	1	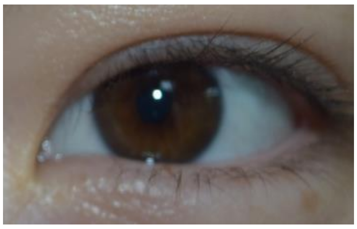
FKZ 21-361	0	0	1	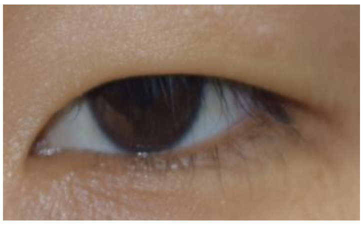
FKZ 21-362	0	0	1	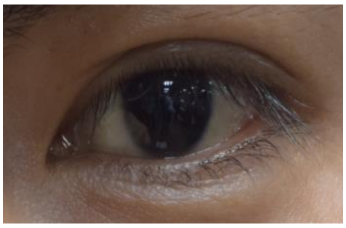
FKZ 21-363	0	1	0	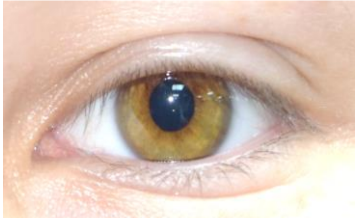
FKZ 21-364	0	0	1	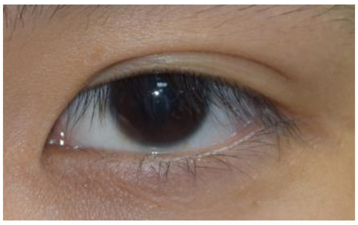
FKZ 21-365	0	0	1	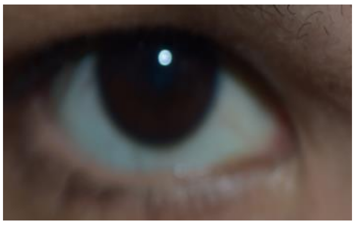
FKZ 21-366	0	0	1	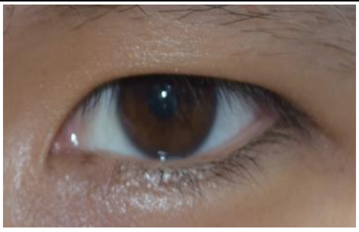

FKZ 21-368	0	0	1	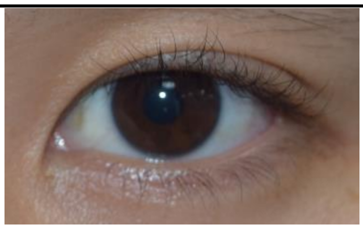
FKZ 21-369	0	0	1	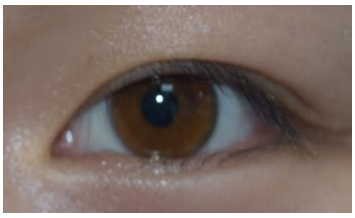
FKZ 21-370	0	0	1	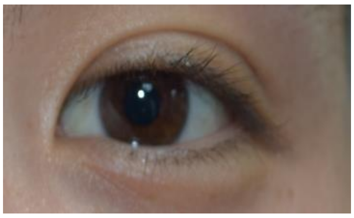
FKZ 21-371	0	0	1	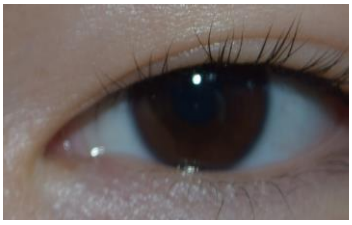
FKZ 21-372	0	0	1	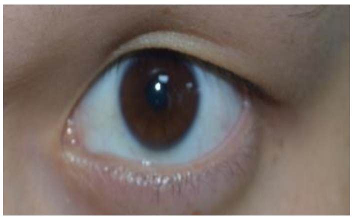
FKZ 21-375	1	0	0	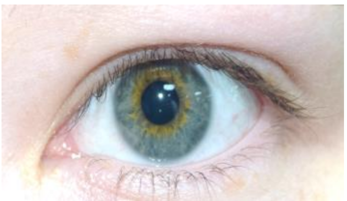
FKZ 21-376	0	0	1	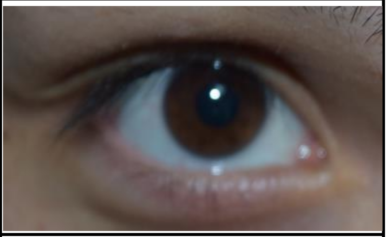
FKZ 21-377	0	0	1	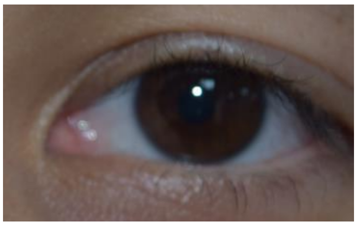

FKZ 21-378	0	0	1	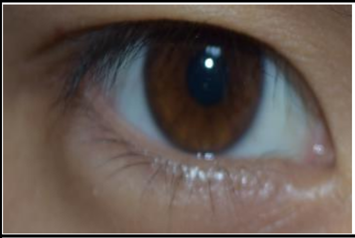
FKZ 21-380	0	0	1	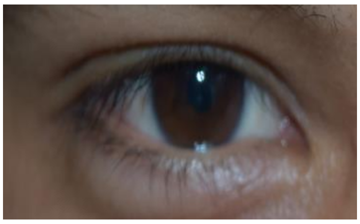
FKZ 21-381	0	0	1	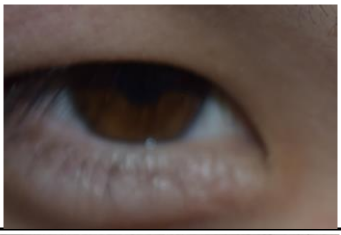
FKZ 21-382	0	0	1	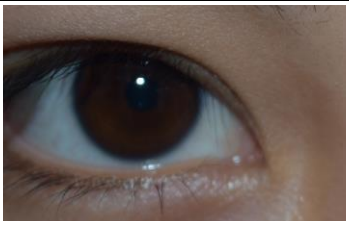
FKZ 21-383	0	0	1	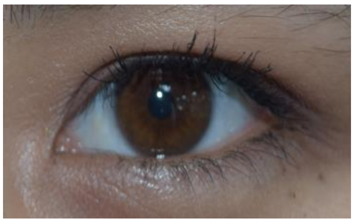
FKZ 21-384	0	0	1	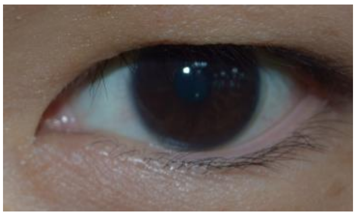
FKZ 21-386	0	0	1	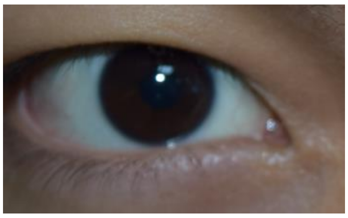
FKZ 21-387	0	0	1	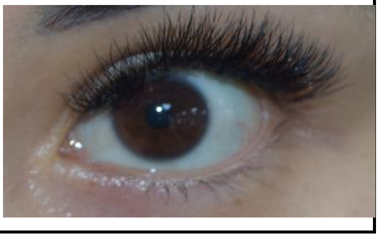

FKZ 21-388	0	0	1	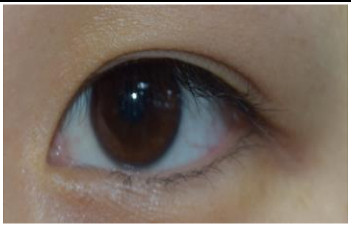
FKZ 21-389	0	0	1	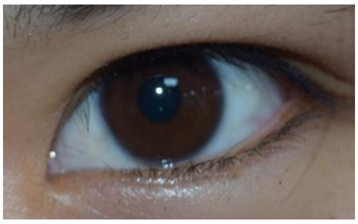
FKZ 21-390	0	0	1	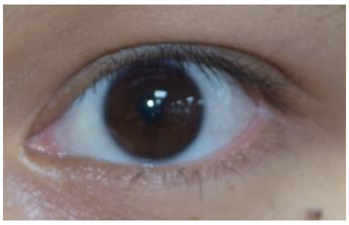
FKZ 21-391	0	0	1	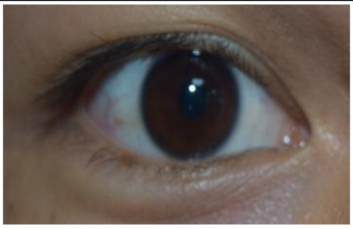
FKZ 21-392	0	0	1	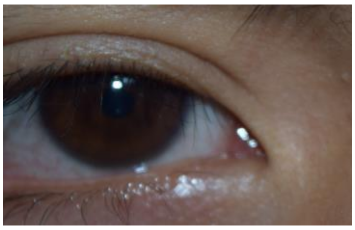
FKZ 21-393	0	0	1	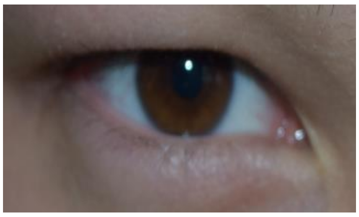
FKZ 21-394	0	0	1	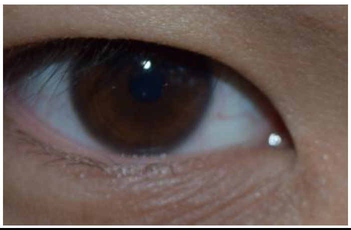
FKZ 21-395	0	0	1	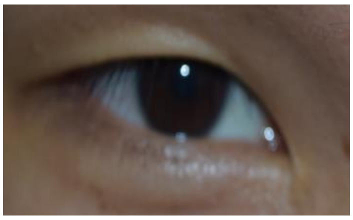

FKZ 21-397	0	0	1	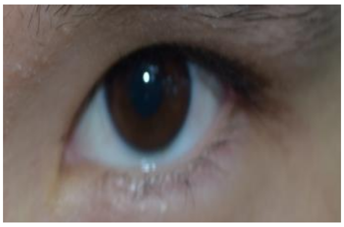
FKZ 21-398	0	1	0	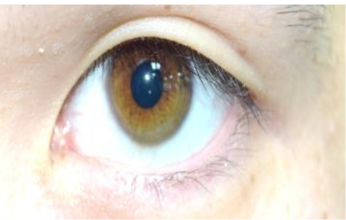
FKZ 21-399	0	0	1	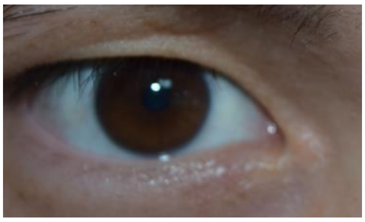
FKZ 21-400	0	0	1	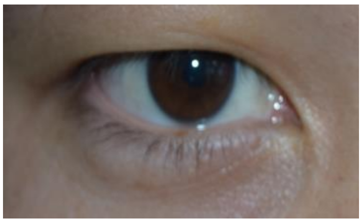
FKZ 21-401	0	0	1	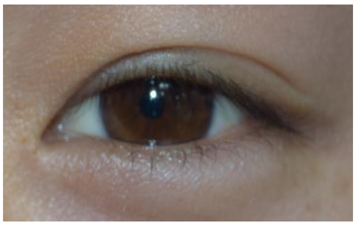
FKZ 21-402	0	0	1	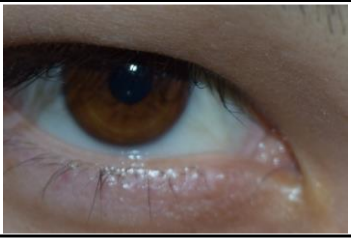
FKZ 21-403	0	0	1	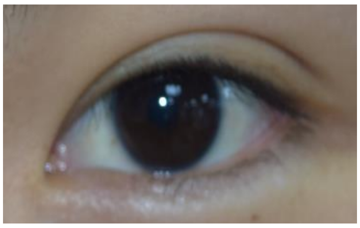
FKZ 21-404	0	0	1	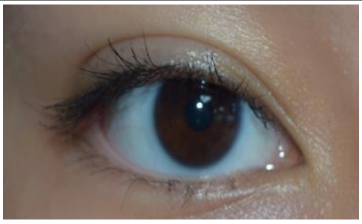

FKZ 21-405	0	0	1	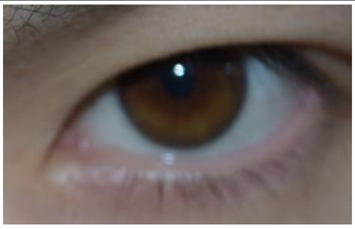
FKZ 21-406	0	0	1	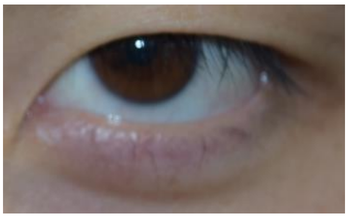
FKZ 21-407	0	0	1	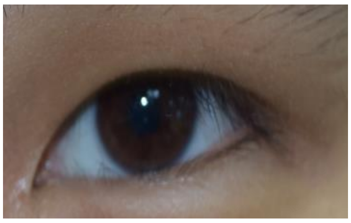
FKZ 21-408	0	1	0	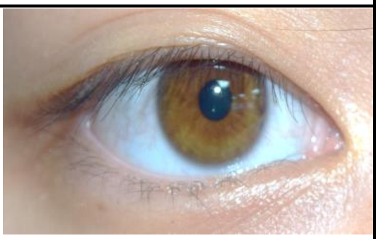
FKZ 21-409	0	0	1	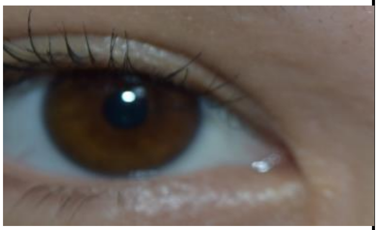
FKZ 21-410	0	0	1	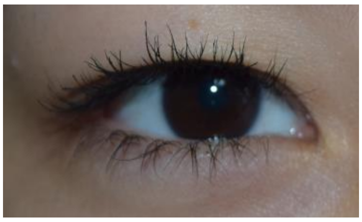
FKZ 21-412	0	0	1	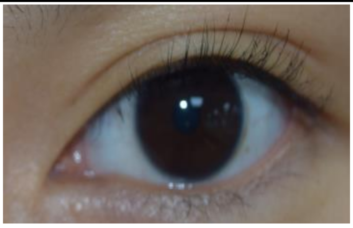
FKZ 21-413	0	0	1	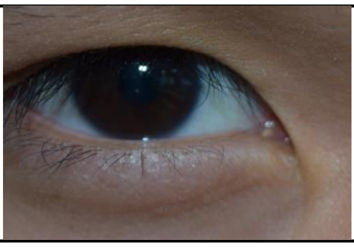

FKZ 21-414	0	0	1	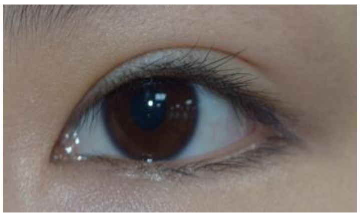
FKZ 21-417	0	0	1	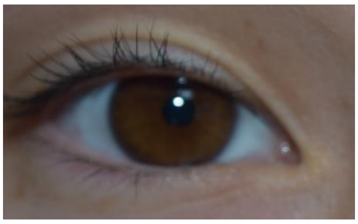
FKZ 21-418	0	0	1	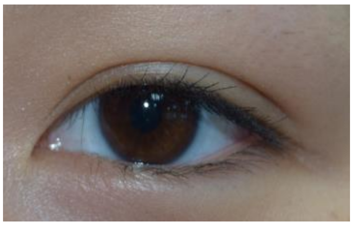
FKZ 21-419	0	0	1	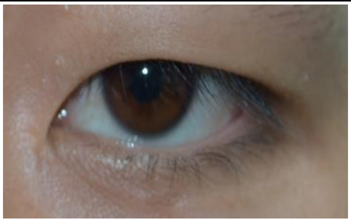
FKZ 21-420	0	1	0	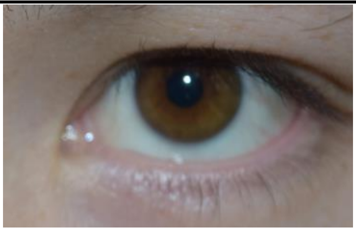
FKZ 21-421	0	0	1	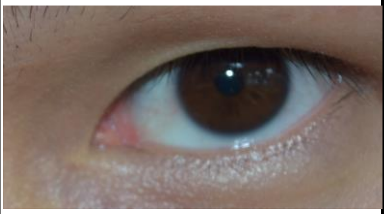
FKZ 21-425	0	0	1	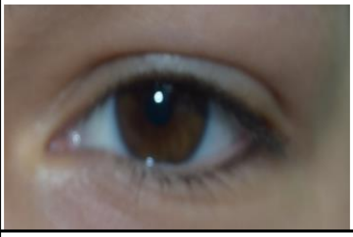
FKZ 21-426	0	0	1	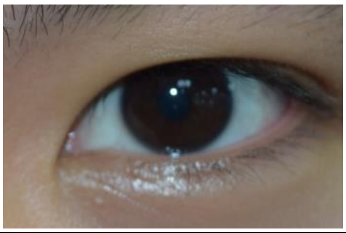

FKZ 21-427	0	0	1	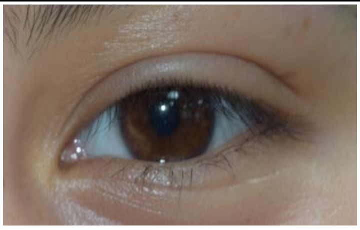
FKZ 21-428	0	0	1	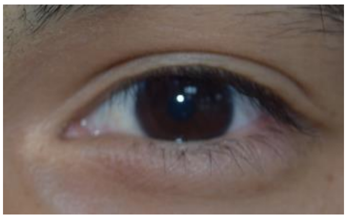
FKZ 21-431	0	0	1	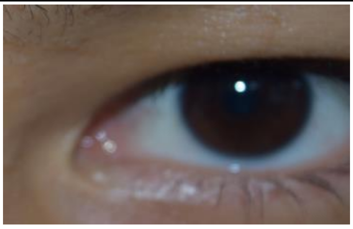
FKZ 21-432	0	1	0	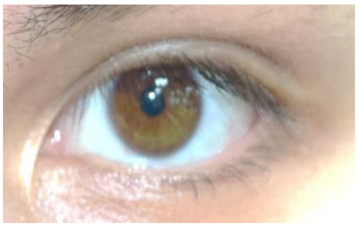
FKZ 21-433	0	0	1	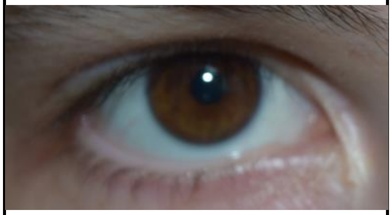
FKZ 21-434	0	0	1	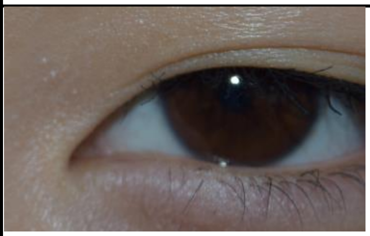
FKZ 21-435	0	0	1	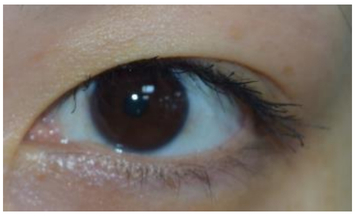
FKZ 21-436	0	0	1	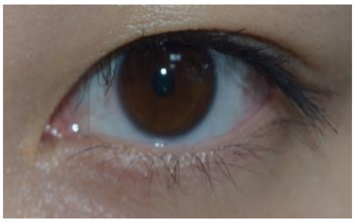

FKZ 21-437	0	0	1	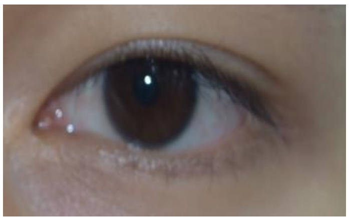
FKZ 21-438	0	0	1	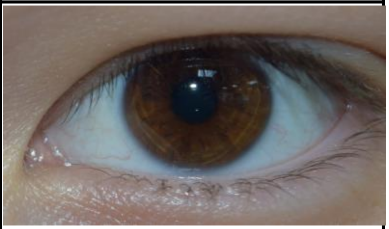
FKZ 21-440	0	1	[illegible]	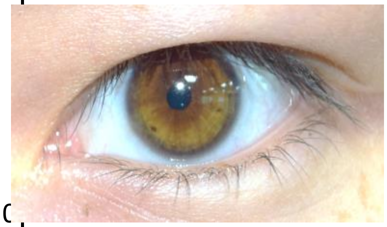
FKZ 21-441	0	0	1	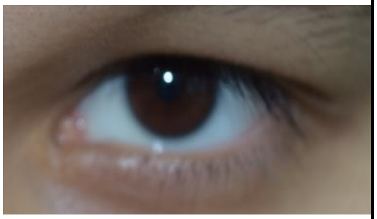
FKZ 21-442	0	0	1	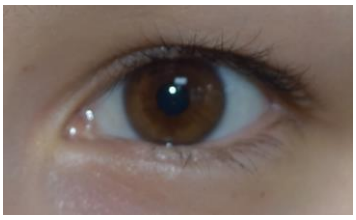
FKZ 21-443	0	0	1	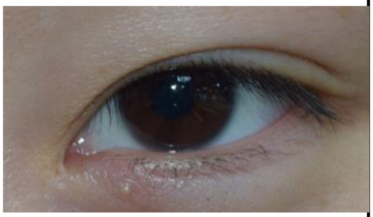
FKZ 21-445	0	0	1	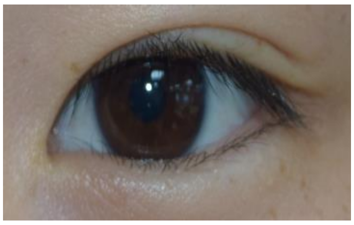
FKZ 21-446	0	1	0	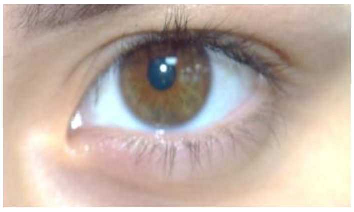

FKZ 21-447	0	0	1	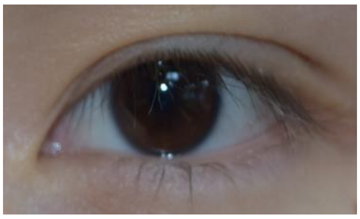
FKZ 21-448	0	1	0	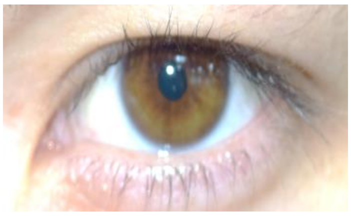
FKZ 21-449	0	0	1	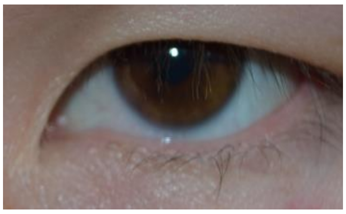
FKZ 21-451	0	0	1	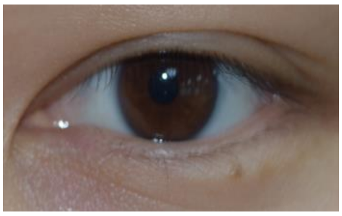
FKZ 21-452	0	0	1	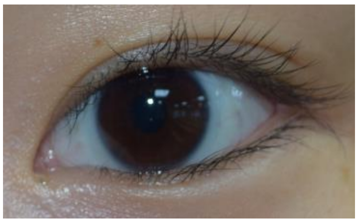
FKZ 21-453	0	0	1	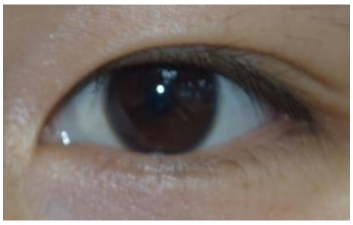
FKZ 21-455	0	0	1	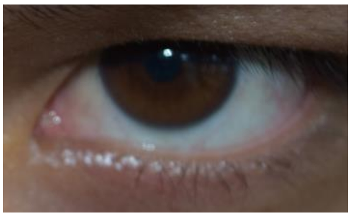
FKZ 21-456	0	0	1	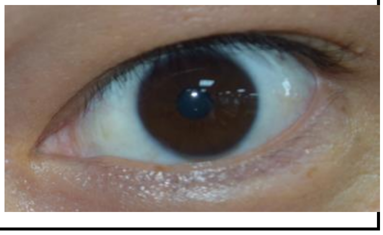

FKZ 21-457	0	0	1	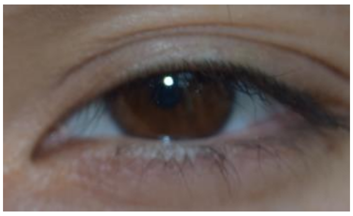
FKZ 21-458	0	0	1	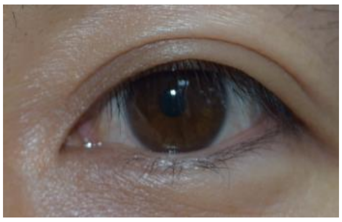
FKZ 21-459	0	1	0	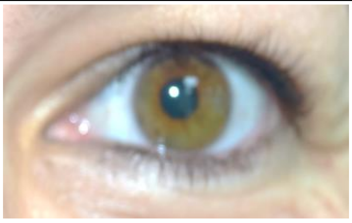
FKZ 21-460	0	0	1	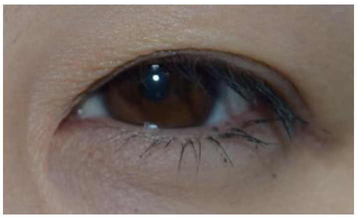
FKZ 21-461	0	0	1	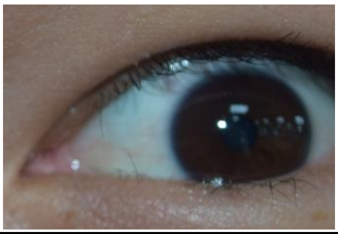
FKZ 21-462	0	0	1	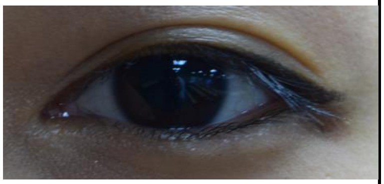
FKZ 21-463	0	1	0	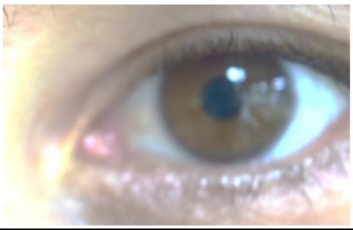
FKZ 21-465	0	0	1	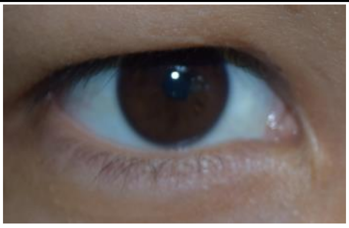

FKZ 21-466	0	0	1	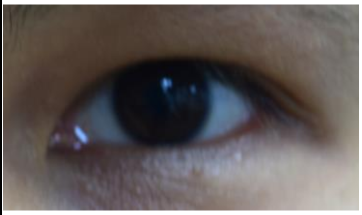
FKZ 21-467	0	0	1	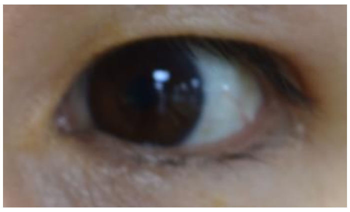
FKZ 21-468	0	0	1	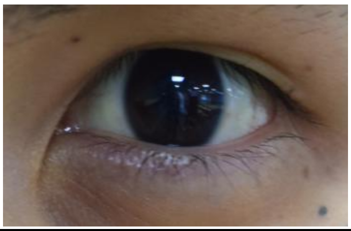
FKZ 21-471	0	0	1	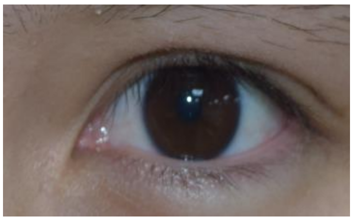
FKZ 21-475	0	0	1	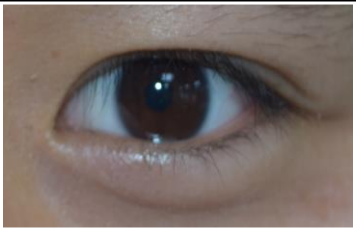
FKZ 21-476	0	0	1	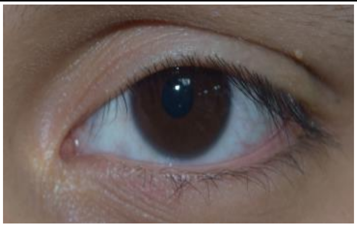
FKZ 21-479	0	0	1	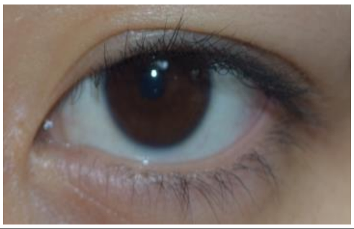
FKZ 21-482	0	0	1	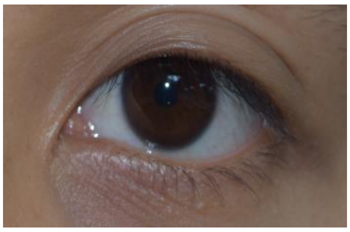

FKZ 21-485	0	0	1	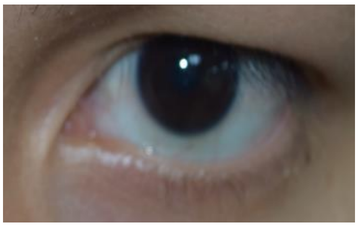
FKZ 21-486	0	0	1	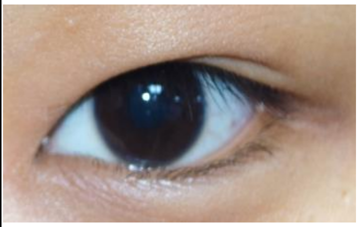
FKZ 21-487	0	1	0	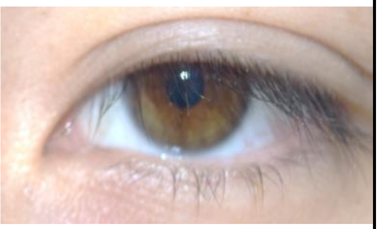
FKZ 21-489	0	0	1	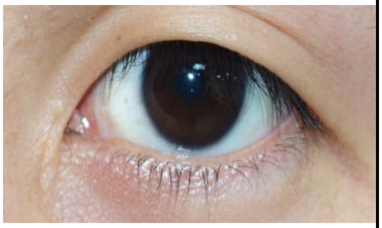
FKZ 21-490	0	1	0	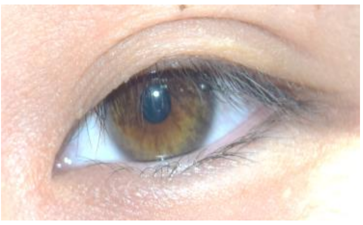
FKZ 21-491	0	0	1	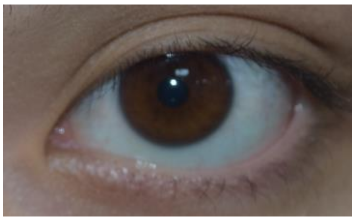
FKZ 21-492	0	0	1	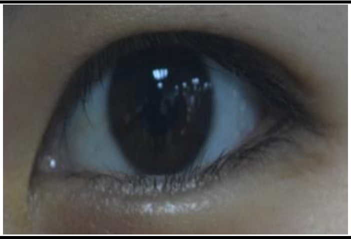
FKZ 21-493	0	0	1	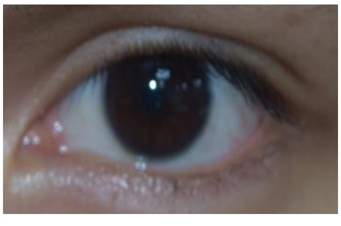

FKZ 21-494	0	1	0	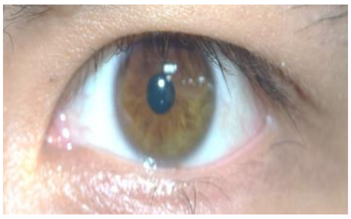
FKZ 21-496	0	0	1	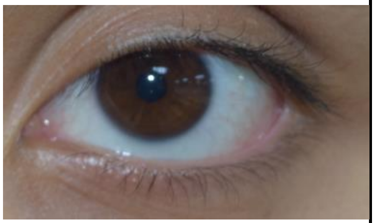
FKZ 21-497	0	0	1	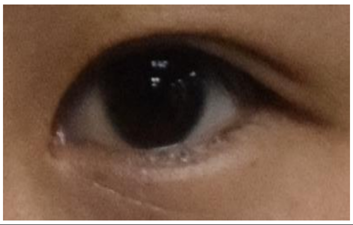
FKZ 21-498	0	0	1	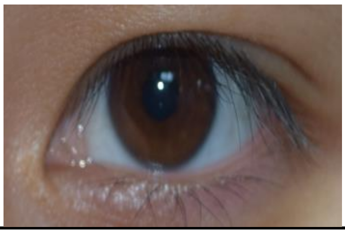
FKZ 21-499	1	0	0	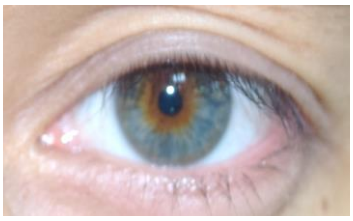
FKZ 21-500	0	0	1	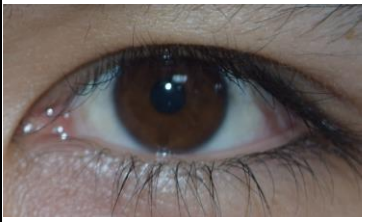
FKZ 21-501	0	1	0	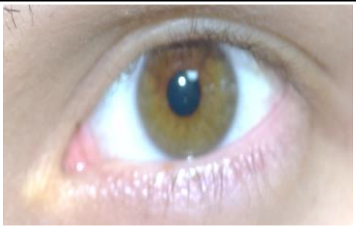
FKZ 21-504	0	0	1	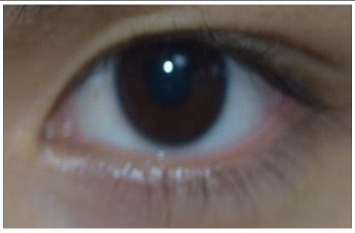

FKZ 21-505	0	0	1	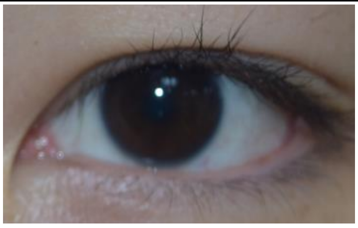
FKZ 21-506	0	1	0	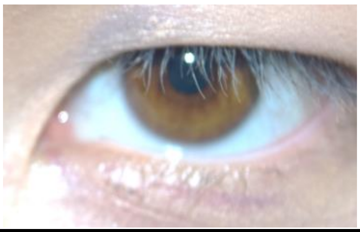
FKZ 21-507	0	0	1	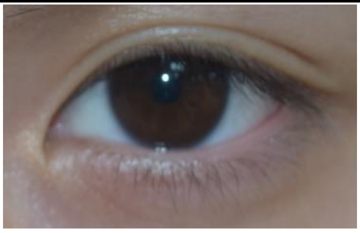
FKZ 21-508	0	0	1	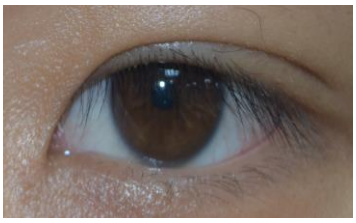
FKZ 21-509	1	0	0	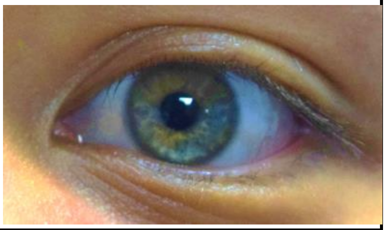
FKZ 21-510	0	1	0	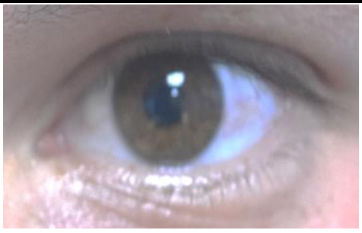
FKZ 21-511	0	0	1	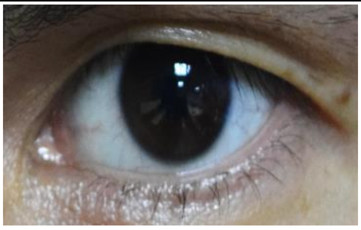
FKZ 21-512	0	1	0	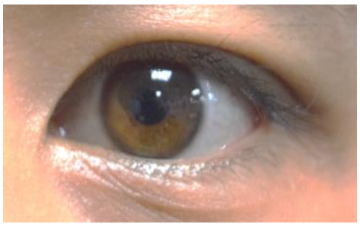

FKZ 21-513	0	0	1	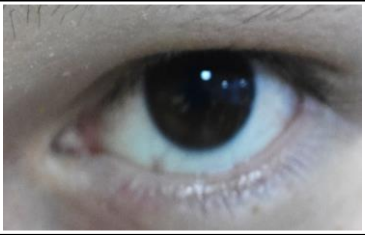
FKZ 21-514	0	0	1	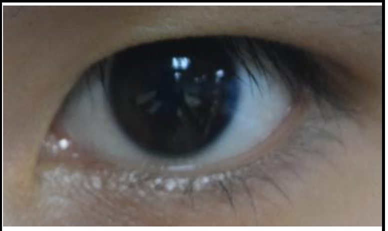
FKZ 21-515	0	0	1	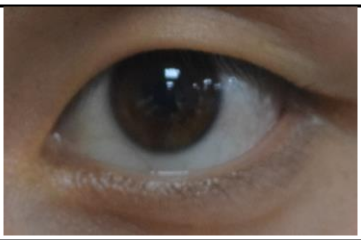
FKZ 21-517	0	0	1	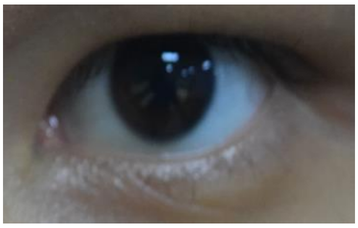
FKZ 21-518	0	0	1	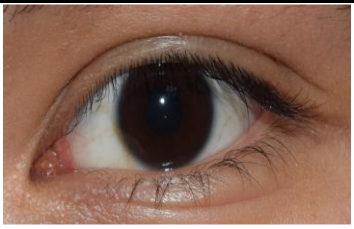
FKZ 21-519	0	0	1	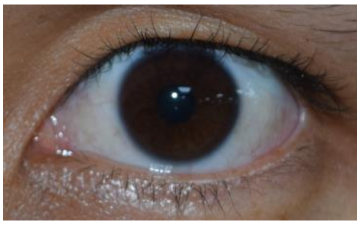
FKZ 21-520	0	1	0	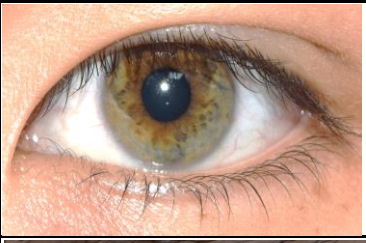
FKZ 21-522	0	0	1	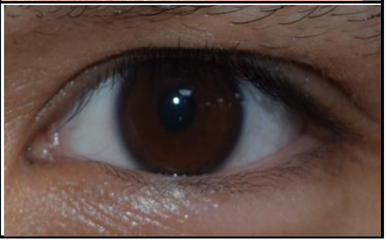

FKZ 21-523	0	0	1	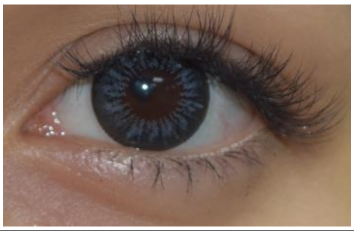
FKZ 21-524	0	0	1	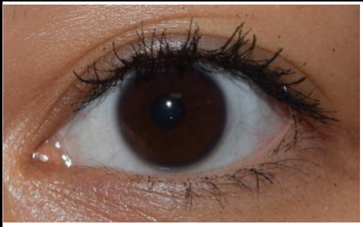
FKZ 21-525	0	0	1	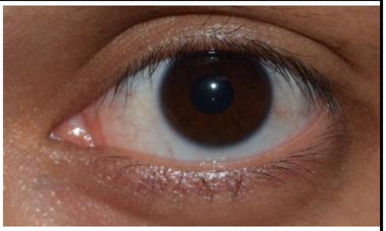
FKZ 21-527	0	0	1	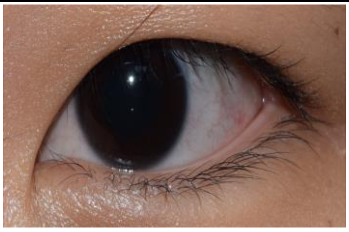
FKZ 21-528	0	0	1	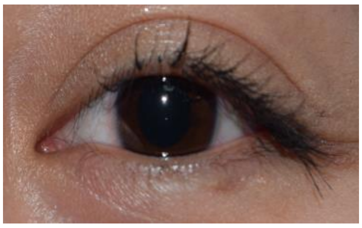
FKZ 21-529	0	0	1	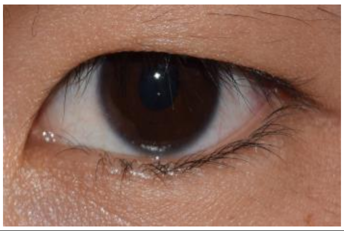
FKZ 21-530	0	0	1	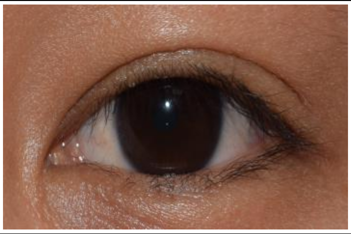
FKZ 21-531	0	0	1	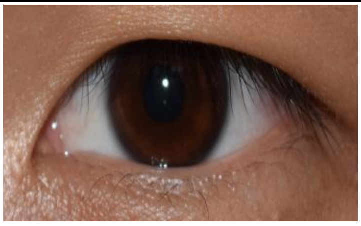

FKZ 22-001	0	1	0	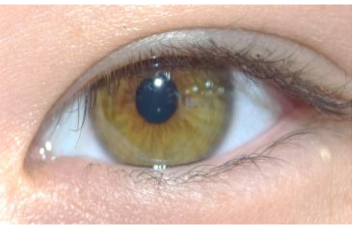
FKZ 22-002	0	0	1	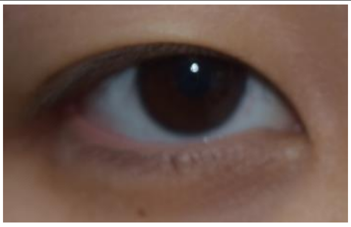
FKZ 22-007	0	0	1	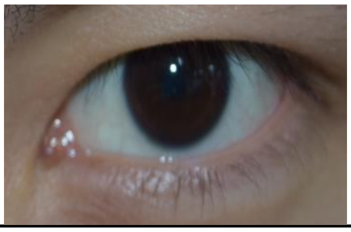
FKZ 22-008	0	1	0	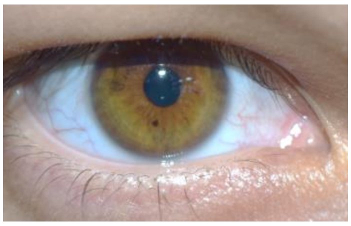
FKZ 22-009	0	0	1	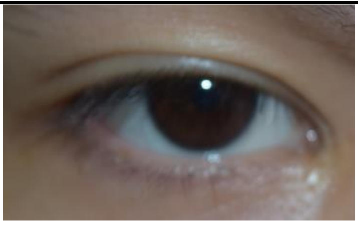
FKZ 22-010	0	0	1	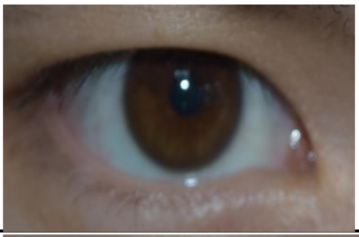
FKZ 22-011	0	0	1	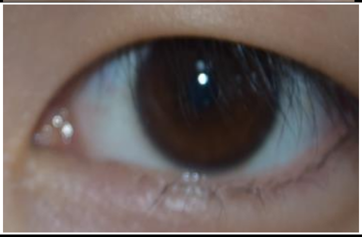
FKZ 22-013	0	1	0	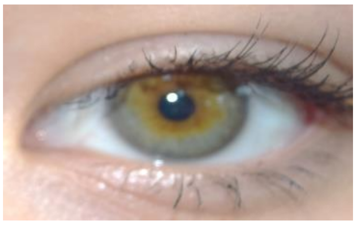

FKZ 22-014	0	0	1	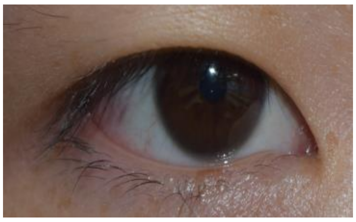
FKZ 22-015	0	0	1	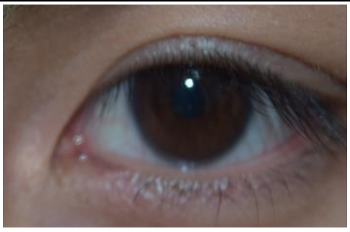
FKZ 22-020	0	0	1	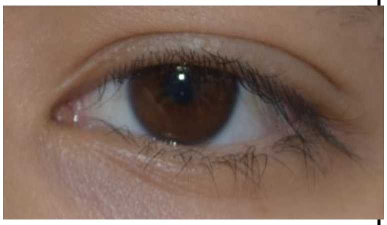
FKZ 22-021	0	0	1	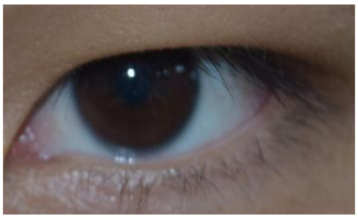
FKZ 22-023	0	0	1	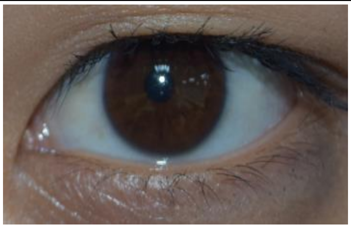
FKZ 22-024	0	0	1	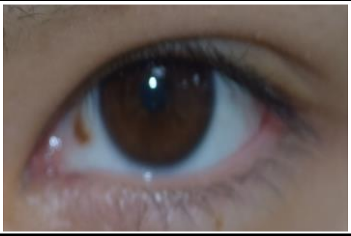
FKZ 22-026	0	1	0	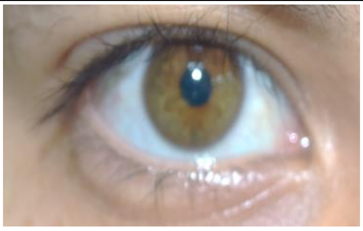
FKZ 22-027	0	0	1	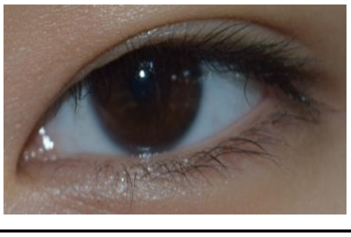

FKZ 22-028	0	0	1	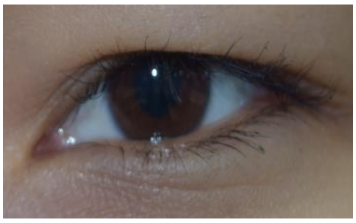
FKZ 22-029	0	0	1	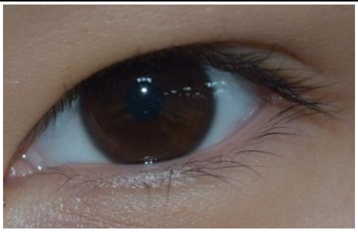
FKZ 22-030	0	1	0	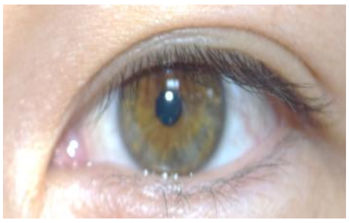
FKZ 22-031	0	0	1	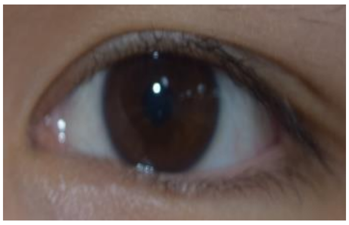
FKZ 22-032	0	0	1	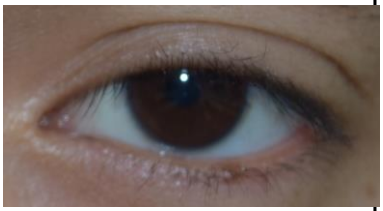
FKZ 22-033	0	0	1	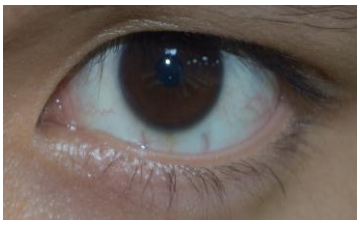
FKZ 22-034	0	0	1	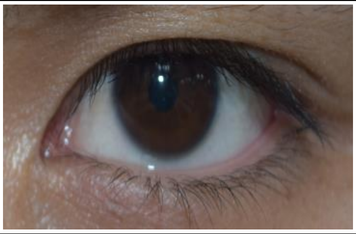
FKZ 22-035	0	0	1	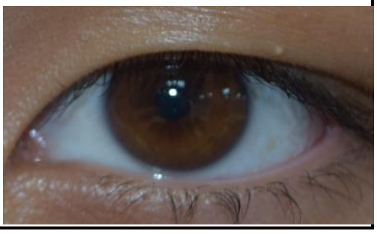

FKZ 22-036	0	0	1	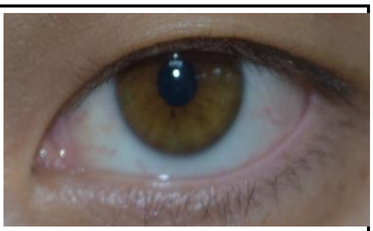
FKZ 22-037	0	0	1	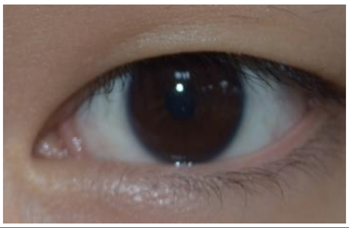
FKZ 22-038	0	0	1	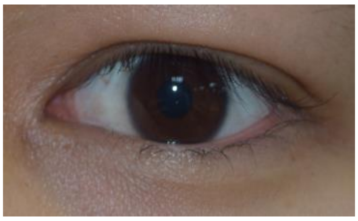
FKZ 22-039	0	0	1	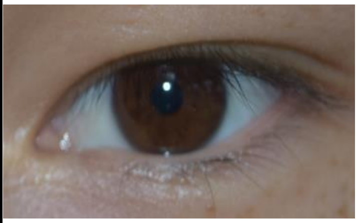
FKZ 22-040	0	0	1	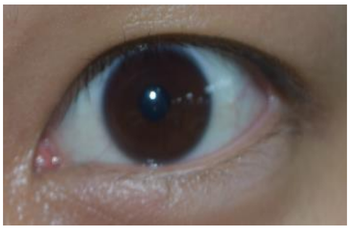
FKZ 22-043	0	0	1	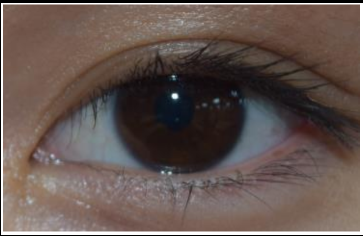
FKZ 22-044	0	0	1	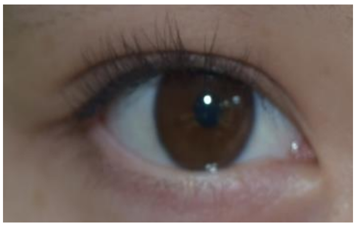
FKZ 22-045	0	0	1	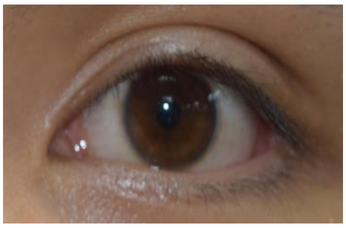

FKZ 22-046	0	0	1	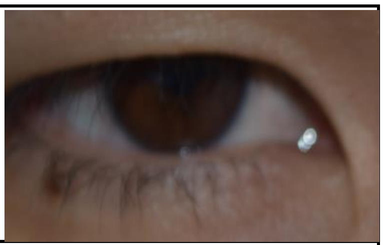
FKZ 22-047	0	1	0	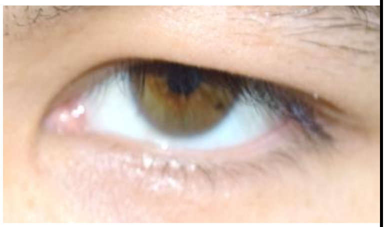
FKZ 22-048	0	0	1	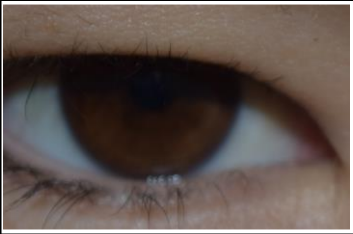
FKZ 22-049	0	0	1	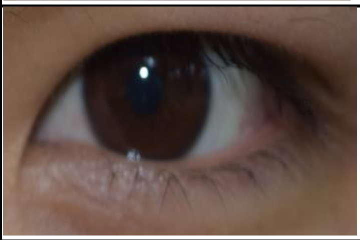
FKZ 22-050	0	0	1	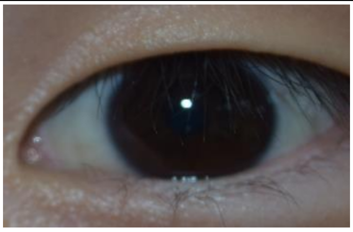
FKZ 22-051	0	0	1	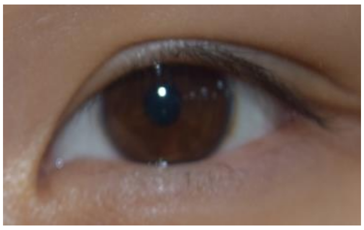
FKZ 22-052	0	0	1	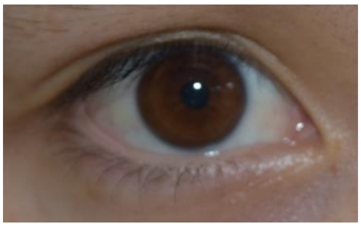
FKZ 22-053	0	0	1	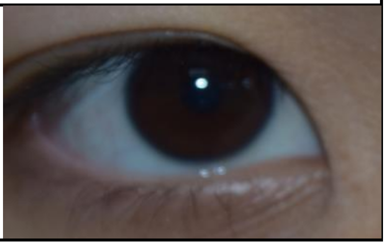

FKZ 22-054	0	0	1	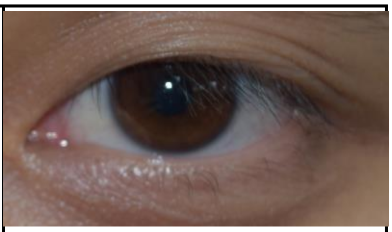
FKZ 22-055	0	0	1	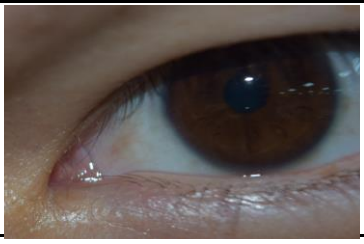
FKZ 22-056	0	0	1	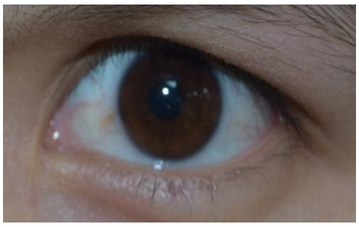
FKZ 22-057	0	0	1	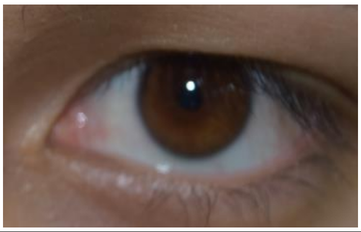
FKZ 22-058	0	0	1	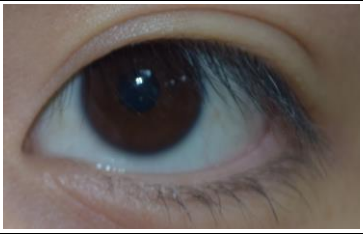
FKZ 22-059	0	0	1	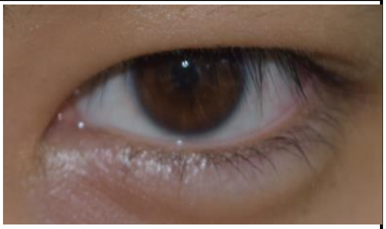
FKZ 22-060	0	1	0	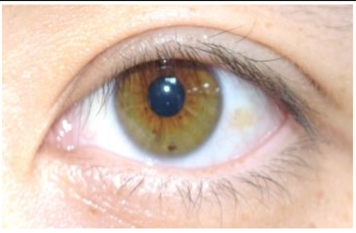
FKZ 22-061	0	0	1	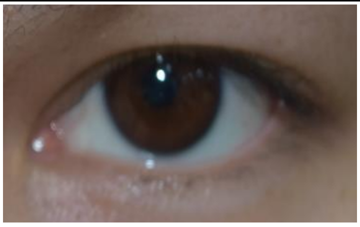

FKZ 22-062	0	0	1	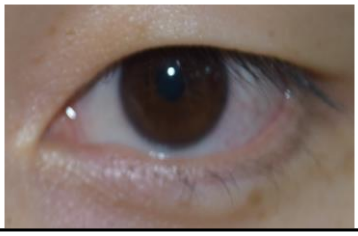
FKZ 22-063	0	0	1	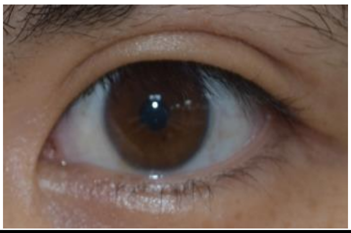
FKZ 22-064	0	0	1	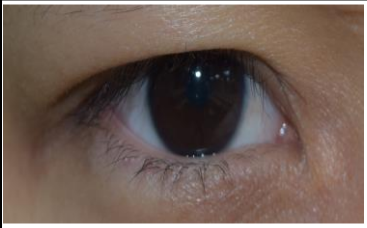
FKZ 22-065	0	0	1	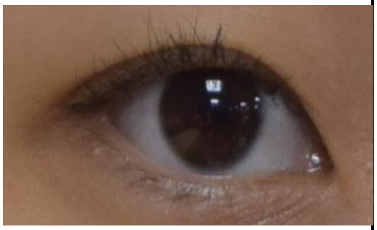
FKZ 22-066	0	0	1	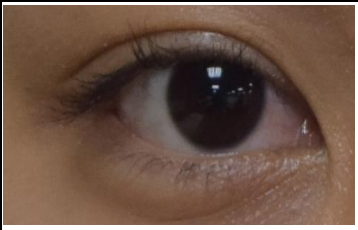
FKZ 22-067	0	0	1	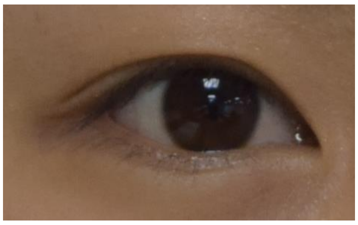
FKZ 22-070	0	0	1	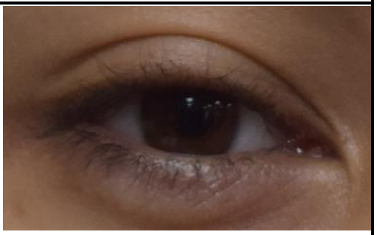
FKZ 22-074	0	0	1	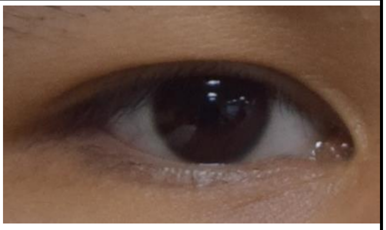

FKZ 22-077	0	0	1	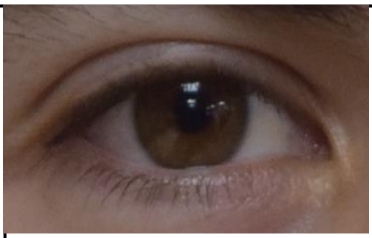
FKZ 22-078	0	0	1	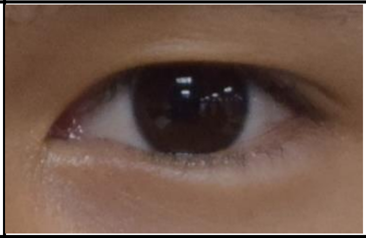
FKZ 22-079	0	0	1	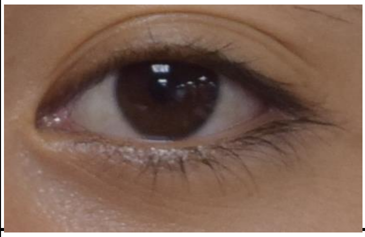
FKZ 22-080	0	0	1	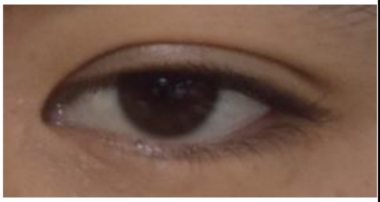
FKZ 22-082	0	0	1	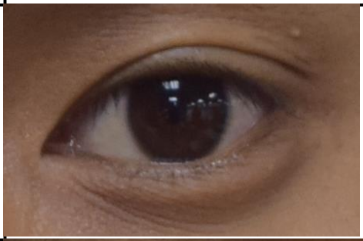
FKZ 22-087	0	0	1	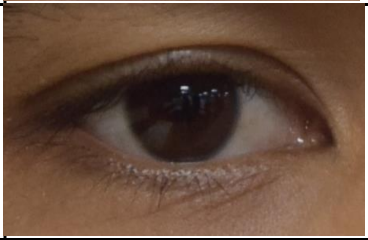
FKZ 22-089	0	0	1	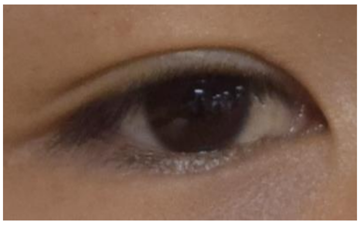
FKZ 22-091	0	0	1	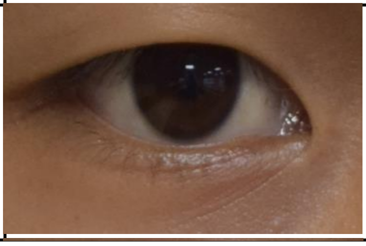

FKZ 22-094	0	0	1	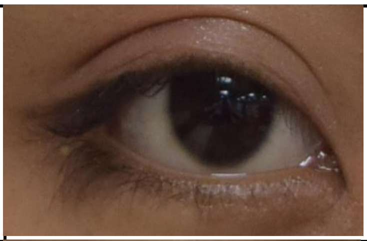
FKZ 22-095	0	0	1	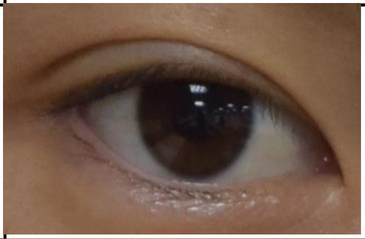
FKZ 22-096	0	0	1	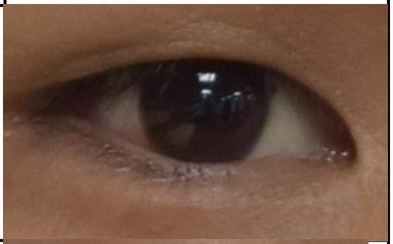
FKZ 22-097	0	0	1	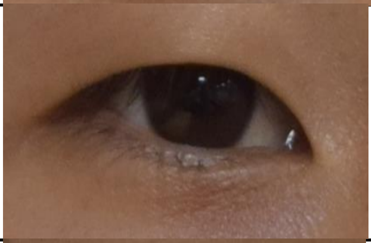
FKZ 22-098	0	0	1	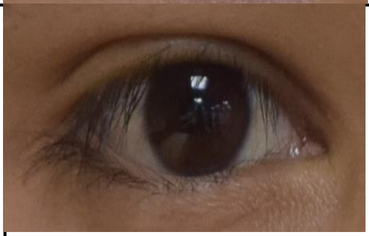
FKZ 22-100	0	0	1	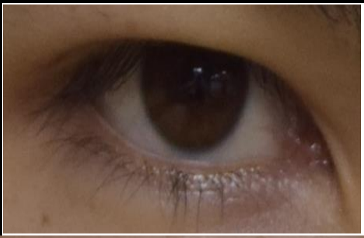
FKZ 22-101	0	0	1	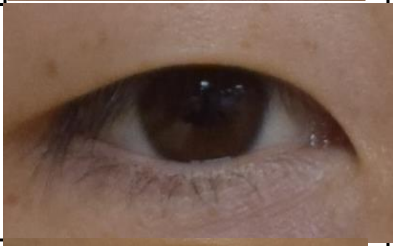
FKZ 22-102	0	0	1	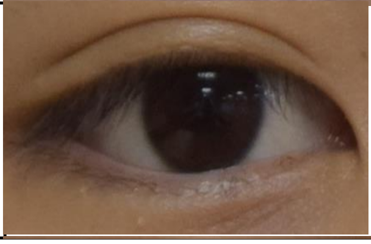

FKZ 22-103	0	0	1	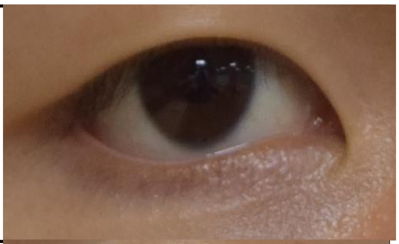
FKZ 22-104	0	0	1	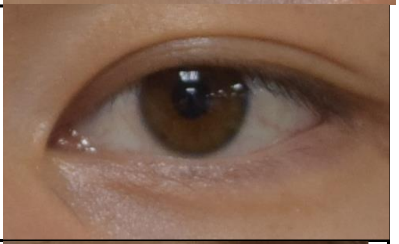
FKZ 22-105	0	0	1	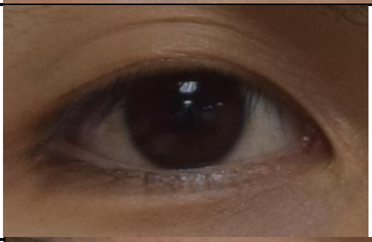
FKZ 22-107	0	0	1	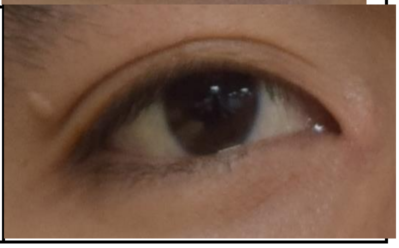
